# Allosteric Inhibition of Polycomb Repressive Complex 2 by an EZH2‐Selective Small Molecule Inhibitor

**DOI:** 10.1002/advs.76025

**Published:** 2026-06-18

**Authors:** Ting Cao, Dongdong Liu, Haishan Gao, Chenyang Qin, Wen Zhang, Ziyun Lu, Dongxia Tan, Yuxiu Qu, Yusong Liu, Zhiran Zou, Hongtao Yu, Wei Qi

**Affiliations:** ^1^ Gene Editing Center, School of Life Science and Technology ShanghaiTech University Shanghai China; ^2^ Westlake Laboratory of Life Sciences and Biomedicine, School of Life Sciences Westlake University Hangzhou Zhejiang China; ^3^ New Cornerstone Science Laboratory Westlake University Hangzhou Zhejiang China; ^4^ Shanghai Clinical Research and Trial Center Shanghai China; ^5^ Lingang Laboratory Shanghai China

**Keywords:** allosteric mechanism, cancer immunotherapy, EZH2 inhibitor, PRC2

## Abstract

Polycomb Repressive Complex 2 (PRC2), containing homologous EZH1 or EZH2 as the catalytic subunit, is a conserved methyltransferase complex that represses gene transcription by transferring methyl group from SAM to H3K27. Gain‐of‐function mutations of EZH2 and aberrant H3K27 methylation have been linked to human cancers. SAM‐competitive EZH1 and EZH2 dual inhibitors have been approved by the FDA for treating B‐cell lymphoma and other cancers. Here, we characterized a small molecule C36, which potently inhibits EZH2/PRC2, but not EZH1/PRC2, with a novel SAM non‐competitive mechanism. Cryo‐EM structures revealed that C36 binds to a pocket at the interface of SET‐Activation‐Loop (SAL), stimulation‐responsive motif (SRM), and I‐SET domain of EZH2, and WD40 domain of EED. C36 binding induces conformational changes and disrupts allosteric communication between EZH2 and ligand‐bound EED. C36 efficiently inhibits H3K27 trimethylation and PRC2 target gene expression in tumor cells and xenograft tumors with low hematotoxicity. Multi‐omics analyses employing C36 uncovered the direct regulation of *IFNB1* by EZH2/PRC2. The combined treatment of syngeneic LLC lung cancer with C36 and a PD‐1 antibody significantly enhances anti‐tumor efficacy. Our study identifies a new allosteric mechanism of PRC2 inhibition and paves the way for the development of highly selective EZH2/PRC2 inhibitors for combination therapy.

## Graphical abstract

The study characterizes C36, a highly selective EZH2/PRC2 inhibitor that acts via a novel allosteric mechanism. Unlike previous inhibitors, C36 inhibits EZH2/PRC2 by disrupting the allosteric communication between EZH2 and EED in a SAM‐noncompetitive manner. Consequently, C36 efficiently inhibits H3K27 trimethylation and suppresses tumor growth with low hematotoxicity.

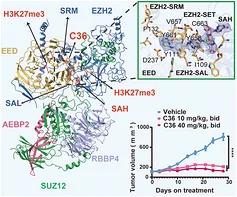

## 1 Introduction

Polycomb Repressive Complex 2 (PRC2) is the sole methyltransferase responsible for mono‐, di‐, and tri‐methylation of histone H3 lysine 27 (H3K27me1/2/3), a critical histone modification involved in transcriptional repression and chromatin compaction. The core subunits of PRC2 include the catalytic subunit EZH2 (or its closely related homolog EZH1), SUZ12, and EED, all of which are critical for proper gene expression during embryonic development. PRC2 mediates the repression of key genes involved in cell cycle regulation and stemness maintenance, such as *CDKN1A*, *HLA‐B*, and *BMPs* [1, 2, 3]. Gain‐of‐function (GOF) mutations and aberrant overexpression of EZH2 have been implicated in various human cancers [4], and drive or facilitate oncogenesis in genetic models [5, 6]. EZH2 expression is regulated by the E2F pathway and is thus linked to cell proliferation [1]. Chemical inhibitors targeting EZH2 and EED have been developed and shown promise in preclinical and clinical studies, with notable efficacy in treating B‐cell lymphoma, malignant rhabdoid tumors, and prostate cancer [7, 8, 9, 10, 11].

**FIGURE 1:**
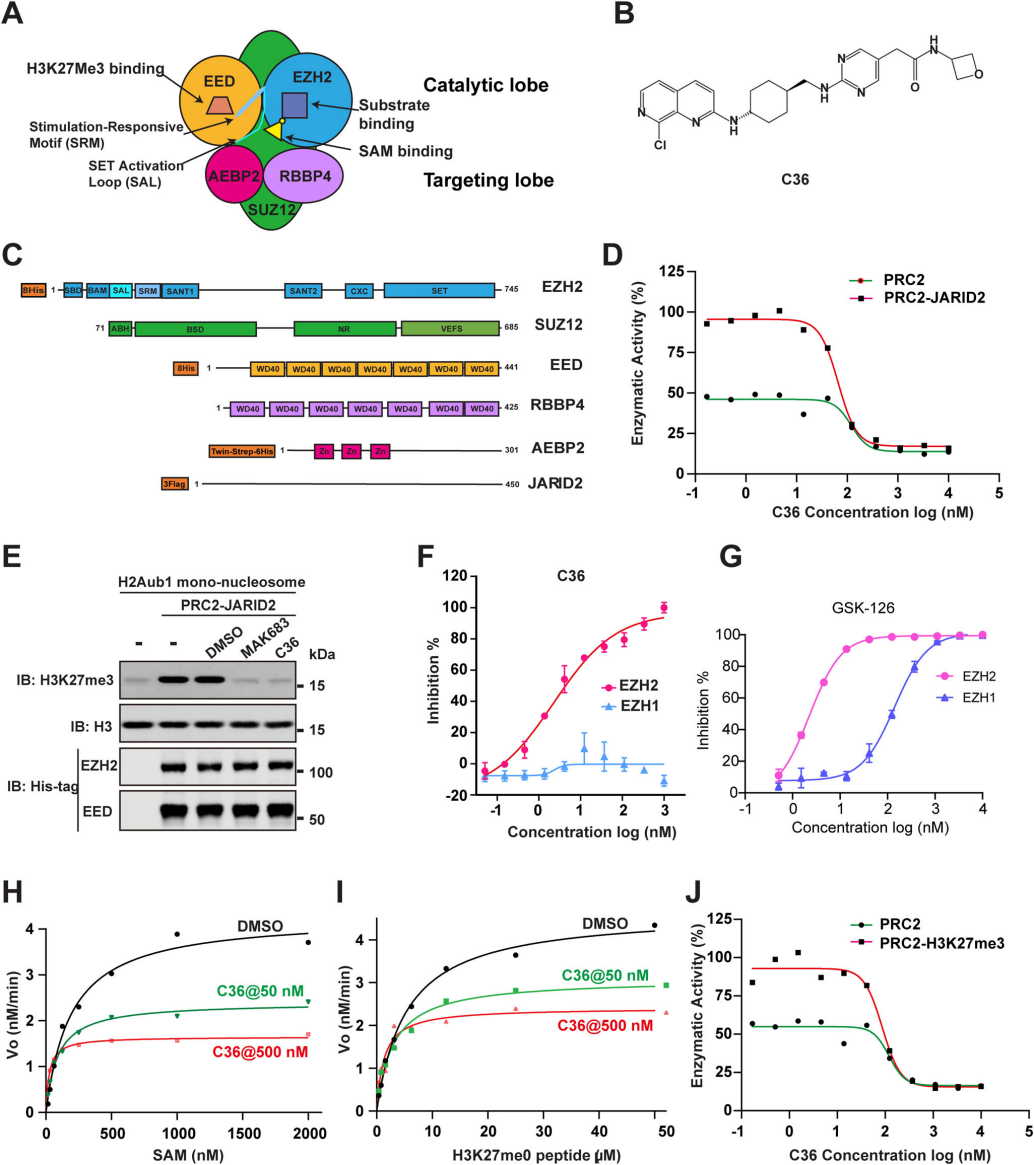
C36 is a unique and selective EZH2‐PRC2 inhibitor. (A) Schematic model of PRC2 complex. (B) C36 chemical formula. (C) Domain schemes of each subunit of human PRC2 used in the current study. (D) In vitro enzymatic inhibition of PRC2, PRC2‐JARID2 by C36. (E) In vitro enzymatic inhibition of PRC2‐JARID2 by C36 on mono‐nucleosome substrate. Repeated 3 times. (F) Effect of C36 on EZH2/PRC2 (IC_50_ = 2.274 nm) and EZH1/PRC2 (IC_50_ > 1000 nm) enzymatic activity as measured by AlphaLISA assay. (G) Effect of GSK126 on EZH2/PRC2 (IC_50_ = 2.258 nm) and EZH1/PRC2 (IC_50_ = 141.1 nm) enzymatic activity as measured by AlphaLISA assay. (H) Enzymatic competition assays between SAM and C36. (I) Enzymatic competition assays between H3K27me0 substrate peptide and C36. (J) Enzymatic competition assays between H3K27me3 substrate peptide and C36.

The PRC2 complex exists in two forms with mutually exclusive accessory subunits. In addition to the core subunits and RBBP4/7, PRC2.1 contains EPOP, PALI1/2, and one of the Polycomb‐like (PCL) proteins while PRC2.2 contains AEBP2 and JARID2 [12]. The integrated assembly of the PRC2 complex is essential for its enzymatic activity, regulation by local chromatin, and proper function. EED allosterically activates EZH2 within the PRC2 complex through binding to H3K27me3 or methylated JARID2 (Figure 1A) [13, 14]. SUZ12 bridges the enzymatic core with AEBP2, JARID2, and PCL proteins, which regulate PRC2 activity and its recruitment to chromatin [15].

Recent structural studies have provided detailed insights into the molecular mechanisms underlying PRC2 assembly and activity (Figure 1A) [16]. The catalytic SET domain of EZH2 alone is in an auto‐inhibited state. In the active PRC2 complex, multiple regions of EZH2 interact extensively with EED and SUZ12. In this way, the autoinhibition of EZH2 is released, exposing the substrate‐binding pocket located in the catalytic lobe [17, 18, 19]. Furthermore, the binding of H3K27me3 to EED causes a conformational change of the stimulation‐responsive motif (SRM) in EZH2, leading to enhanced catalytic efficiency [14]. Spatially apart from the catalytic lobe, RBBP4 and the N‐terminal region of SUZ12 form the targeting lobe, which harbors the surfaces for interaction with PRC2.1 and PRC2.2 specific accessory proteins [16, 20]. Cryo‐EM studies have also revealed detailed interactions between PRC2 and chromatin. For example, the EZH2‐CXC domain interacts with the substrate nucleosome DNA, whereas a lysine patch within EED interacts with DNA in the nucleosome that acts as the allosteric activator of PRC2 [21]. Collectively, these structural studies have established the mechanisms of the catalysis, allosteric regulation, and chromatin engagement by PRC2.

Two classes of small molecule inhibitors targeting PRC2 have been reported (Figure S1) [7, 8, 9, 22, 23, 24, 25]. The first class consists of EZH1 and EZH2‐binding SAM‐competitive inhibitors, such as tazemetostat [7] (also known as EPZ6438 and TAZVERIK) and valemetostat [22]. Crystal structures of PRC2 in complex with this class of compounds showed that their binding site partially overlaps with that of SAM, explaining their competition with SAM [26]. The second class of PRC2 inhibitors includes EED‐binding molecules, such as EED226 [23], MAK683 [8], and HJM353 [27]. They directly bind to the WD40 domain of EED and allosterically inhibit the enzymatic activity of EZH1 and EZH2, while also blocking the binding of H3K27me3 or methylated JARID2 to EED [23]. These compounds have shown therapeutic potential in treating lymphoma and other cancers in clinical trials, although they have not yet received regulatory approval. With the recent research progress in the function of EZH1, it is increasingly clear that EZH1 may not only compensate EZH2 in early embryonic development, but also play critical roles in neurogenesis and circadian regulation in post‐mitotic tissues. Therefore, a highly selective EZH2/PRC2 inhibitor without EZH1/PRC2 inhibition may provide a cleaner safety profile and a better option in a drug‐combinatory cancer regimen.

In this study, we characterize the mechanism of action of the small molecule C36, a potent inhibitor of PRC2. Using biochemical, structural, and functional assays, we demonstrate that C36 targets and inhibits both the basal and activated forms of the PRC2 complex containing EZH2, but not EZH1, in a SAM‐noncompetitive manner. Unlike EED binders, C36 acts through a new allosteric mechanism by binding to a pocket on EZH2 that is adjacent to the SAM‐binding site. This binding induces local conformational changes that disrupt the allosteric communication between EED and EZH2 without affecting the binding of the activating ligand to EED. The EZH2‐selective inhibition by C36 reduced H3K27 methylation levels and upregulated PRC2 target genes in tumor cells, suppressing their proliferation. In a mouse xenograft model, C36 significantly slowed tumor growth, an effect partly attributable to a drastic activation of innate immune signaling. Consequently, combining the PRC2 inhibitors with a PD‐1 antibody significantly enhanced anti‐tumor efficacy in a syngeneic model. Together, these findings provide new insights into the regulatory mechanisms of PRC2 and lay the foundation for developing next‐generation PRC2‐EZH2 inhibitors for cancer combination therapy.

## 2 Results

### 2.1 C36 is a New Class of EZH2‐Selective PRC2 Inhibitor

C36, 2‐(2‐((((1,4‐trans)‐4‐((8‐chloro‐1,7‐naphthyridin‐2‐yl)amino)cyclohexyl)methyl)amino) pyrimidin‐5‐yl)‐N‐(oxetan‐3‐yl)acetamide, is an aza‐quinoline compound for treating EZH2/PRC2 associated cancers initially developed by Novartis [28] (Figure 1B). To investigate the inhibitory mechanism of C36, we expressed and purified recombinant human PRC2 complexes from insect cells and performed biochemical assays (Figure 1C,D). The five‐subunit PRC2 complex (EZH2‐EED‐SUZ12‐RBBP4‐AEBP2) was extensively used in in vitro biochemical assays. PRC2.1 is in the six‐subunit form (EZH2‐EED‐SUZ12‐RBBP4‐AEBP2‐JARID2), in which JARID2 methylated by EZH2 mimics the H3K27me3 peptide and binds to EED to further activate EZH2mimicking [13]. We used both the five‐subunit PRC2 and the six‐subunit PRC2‐JARID2 complexes in our assays. When using the H3K27me0 peptide as a substrate, C36 inhibited the methyltransferase activity of PRC2 and PRC2‐JARID2 with IC_50_ (half‐maximal inhibitory concentration) values of 121 and 66 nm, respectively (Figure 1D). C36 showed incomplete inhibition of PRC2 with the maximum inhibition plateauing at ∼80% (20% PRC2 activity remaining), suggesting that it binds to an allosteric site. C36 also effectively blocked H3K27me3 formation by PRC2‐JARID2 when mono‐nucleosomes with H2AK119 mono‐ubiquitin (H2Aub1) were used as the substrate (Figure 1E). MAK683, an allosteric PRC2 inhibitor that binds to EED, was used as the positive control.

Human EZH1 shares 76% sequence identity with EZH2 (96% in the SET domain). All EED‐binding PRC2 inhibitors are not selective toward EZH1 or EZH2. The SAM‐competitive EZH2 inhibitors are only mildly selective toward EZH2 [9]. C36 showed exceptional selectivity, inhibiting EZH2/PRC2 with an IC_50_ of 2.27 nm, but having no effect on PRC2‐EZH1 (IC_50_>1000 nm) in an AlphaLisa assay (Figure 1F). For comparison, GSK126 only showed a 50‐fold selectivity toward EZH2/PRC2 (Figure 1G). These results indicate that C36 is a potent and highly selective inhibitor of EZH2/PRC2.

We next tested C36 in enzymology studies assessing competition with the cofactor SAM and the substrate H3K27me0 peptide. With increasing SAM concentrations, C36 at 50 and 500 nm decreased the maximum reaction velocity (V_max_) from 4.3 nm/min (DMSO) to 2.4 and 1.7 nm/min, respectively (Figure 1H). High SAM concentrations did not blunt C36 inhibition, suggesting that C36 is not SAM‐competitive. With increasing substrate concentrations, C36 at 50 and 500 nm decreased V_max_ from 4.7 nm/min (DMSO) to 3.1 and 2.4 nm/min (Figure 1I). The K_M_s for SAM and H3K27me0 both decreased in the presence of C36 (Figure S2A,B), indicating that C36 did not compete with SAM or substrate for PRC2 binding. H3K27me3 binding by EED did not significantly affect the IC_50_ of C36 (121 vs 87 nm), suggesting that C36 did not compete with H3K27me3 for EED binding (Figure 1J).

Taken together, C36 is a potent PRC2 inhibitor that is highly selective for EZH2, with a mechanism of action distinct from both SAM‐competitive EZH2 inhibitors and allosteric EED binders. It represents a novel class of PRC2 inhibitors.

### 2.2 C36 Binds to a Novel Allosteric Site on EZH2

**FIGURE 2:**
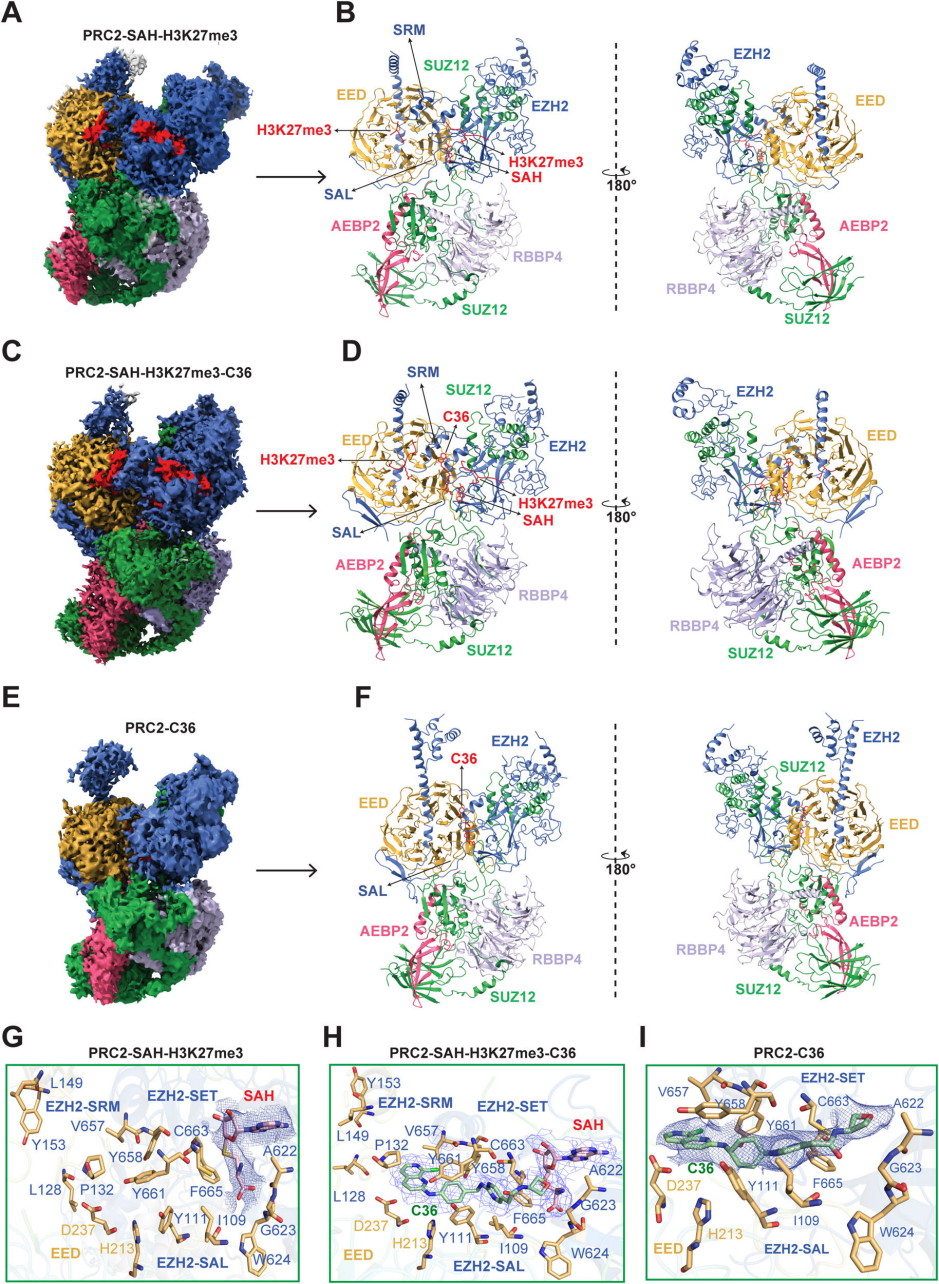
Cryo‐EM structures of PRC2 with C36 bound. (A) Cryo‐EM 3D reconstruction of PRC2‐SAH‐H3K27me3 volume. (B) Structure of PRC2‐SAH‐H3K27me3. (C) Cryo‐EM 3D reconstruction of PRC2‐SAH‐H3K27me3‐C36 volume. (D) Structure of PRC2‐SAH‐H3K27me3‐C36. (E) Cryo‐EM 3D reconstruction of PRC2‐C36 (without peptide, without SAH). (F) Structure of PRC2‐C36. (G) Cryo‐EM density of SAH in the structure of PRC2‐SAH‐H3K27me3. (H) Cryo‐EM density of C36 and SAH in the structure of PRC2‐SAH‐H3K27me3‐C36. (I) Cryo‐EM density of C36 in the structure of PRC2‐C36.

To investigate the structural basis of PRC2 inhibition by C36, we determined the cryo‐EM structures of PRC2 in the basal state bound to C36 (PRC2‐C36) and PRC2 in the activated state with and without C36 (PRC2‐SAH‐H3K27me3‐C36 and PRC2‐SAH‐H3K27me3) (Figure 2A–F). The refined 3D reconstructions, at resolutions ranged from 2.8–3.3 Å, allowed for detailed atomic model building of the five‐subunit PRC2 in its various conformations (Figure 2A–F and Figures S3–S5). PRC2 complexes in all conditions adopted a similar overall architecture, indicating that C36 binding did not drastically alter the global conformation of PRC2 complex (Figure 2C–F). In both PRC2‐SAH‐H3K27me3 and PRC2‐SAH‐H3K27me3‐C36 complexes, the SRM of EZH2 became ordered in the presence of the H3K27me3 peptide (Figure 2A–D). In PRC2‐C36, which did not contain the H3K27me3 peptide, the SRM motif was disordered and less visible (Figure 2E,F). Consistent with previous reports [29, 30], upon H3K27me3 binding, EED stabilizes and anchors the N‐terminal SAL and SRM motifs of EZH2 proximal to the catalytic core, enhancing the activity of EZH2. In both PRC2‐SAH‐H3K27me3 and PRC2‐SAH‐H3K27me3‐C36, a second H3K27me3 peptide molecule bound to the substrate‐binding pocket of EZH2, mimicking the reaction product (Figure 2A–D). In both structures, the EZH2 region C‐terminal to the SET domain became rigid, anchoring the SAH cofactor in the active site of EZH2 (Figure 2A–D).

With the high‐resolution cryo‐EM maps, we were able to accurately place C36 and the cofactor SAH into the density (Figure 2G–I). C36 binds to a pocket that is adjacent to the SAM‐binding site (Figure 2H,I). C36 forms extensive hydrophobic interactions with mainly hydrophobic residues of EED and the I‐SET, SAL, and SRM motifs of EZH2. In particular, the cyclohexyl ring moiety of C36 is sandwiched by the aromatic side chains of Y111, Y658, and Y661 of EZH2 (Figure 2H). I109 and Y111 from SAL and C663 and F665 from I‐SET contact the pyrimidine core of C36 (Figure 2H,I).

The SRM of EZH2 is invisible in the EM map of apo‐PRC2 without H3K27me3 peptide bound [17], but became partially visible in the EM map of the PRC2‐C36 complex (Figure 2I). Thus, C36 binding might stabilize the SRM of EZH2. The oxetan‐3‐yl acetamide group of C36 packs against A622 and C663 of EZH2 in this PRC2 complex without activators (Figure 2I). In contrast, the oxetan‐3‐yl ring loses these interactions but gains interactions with G623 and SAH in the PRC2‐SAH‐H3K27me3‐C36 complex (Figure 2H), suggesting that SAH and C36 may stabilize each other in PRC2 in the activated state. In any case, C36, H3K27me3, and SAH can bind simultaneously to PRC2, consistent with the non‐competitive inhibition mode of C36.

### 2.3 C36 Disrupts the Interaction Network Between EZH2 and EED

**FIGURE 3:**
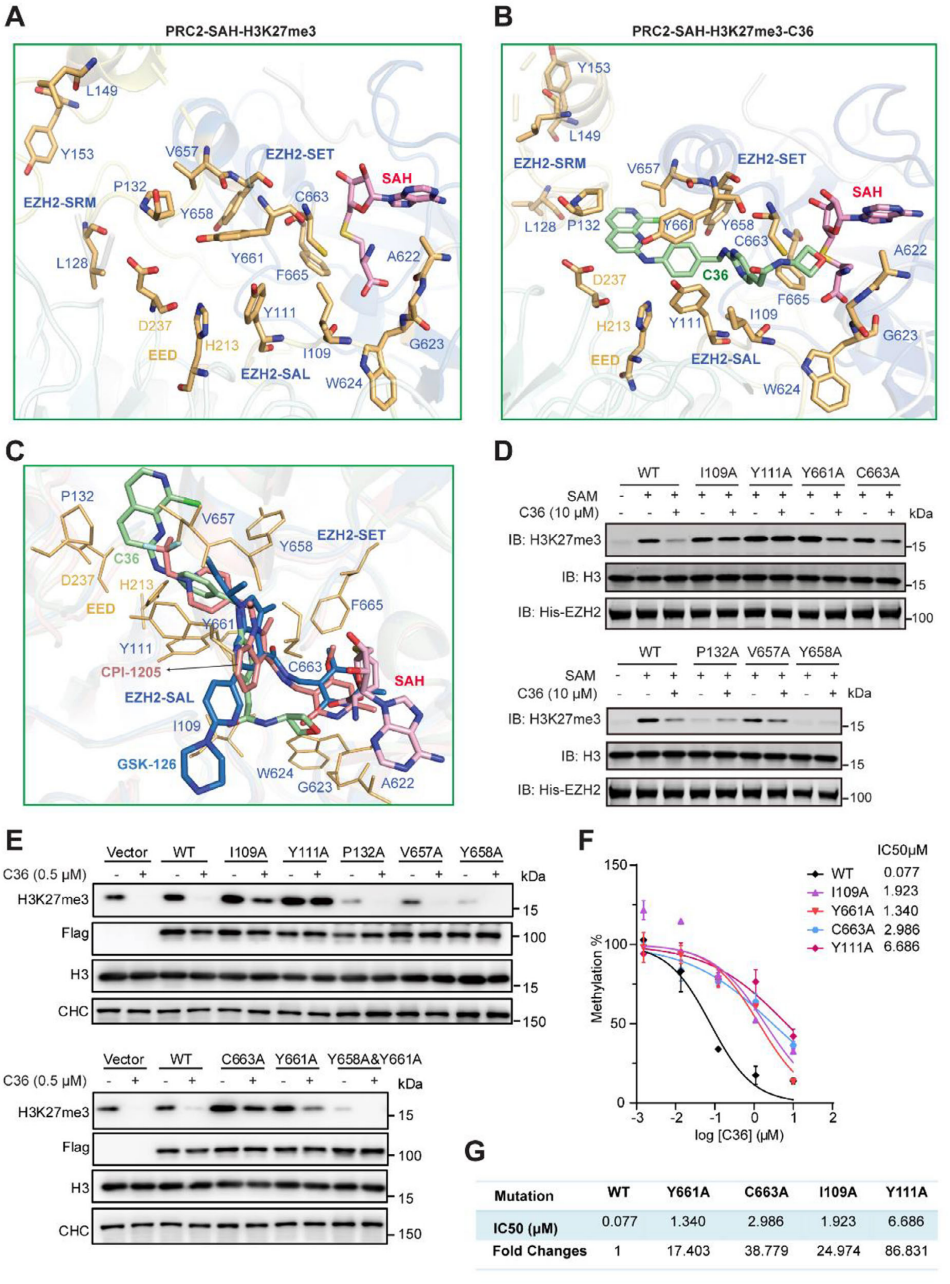
Inhibition mechanism of EZH2/PRC2 by C36. (A) The binding pocket in PRC2‐SAH‐H3K27me3 without C36. (B) The binding pocket in PRC2‐SAH‐H3K27me3 with C36. (C) Structural comparison of C36, GSK‐126, and CPI1205 in PRC2. (D) In vitro biochemical assays for EZH2 mutants with DMSO or C36: I109A, Y111A, Y661A, C663A, P132A, V657A, or Y658A. Repeated 3 times. (E) Western blot analysis was performed to assess the changes in H3K27me3 levels in cells treated with DMSO or C36 for 3 days. G401 cells express EZH2 with I109A, Y111A, P132A, V657A, Y658A, Y661A, C663A, Y658 & Y661A mutations vector and wild type. Repeated 3 times. (F) Representative curves showing the inhibition of H3K27me3 by increasing doses of C36 compound for 3 days. G401 cells express EZH2 with I109A, Y111A, Y661A, C663A mutations and wild type. (G) The table showed the IC_50_ values from panel (F).

To clarify the inhibitory mechanism of C36, we compared the structures of the PRC2‐SAH‐H3K27me3 complex (which represents the activated, post‐catalysis state of PRC2) and the PRC2‐SAH‐H3K27me3‐C36 complex (which is in the C36‐inhibited state). The H3K27me3‐bound EED, SAL, SRM, and I‐SET of EZH2 form an elaborate interaction network in the PRC2‐SAH‐H3K27me3 complex (Figure 3A), consistent with it being in the active state [14, 30]. In this network, V657 from I‐SET contacts P132 of SRM, I109 and Y111 from SAL pack against Y658 and Y661 from I‐SET, forming extensive hydrophobic interactions. Y111 and Y661 also interact with Q191, K211, H213, and K660 of EED (Figure 3A and Figure S6A).

This Y111‐ and Y661‐centered interacting network between SAL, I‐SET, and EED is disrupted by C36 binding (Figure 3B). Specifically, the naphthyridine group of C36 is wedged between P132 and V657, disrupting this hydrophobic contact. H213 and D237 from EED also contact the naphthyridine ring and the neighboring amide group of C36 (Figure 3B and Figure S6B). The cyclohexyl and pyrimidine moiety of C36 are sandwiched between I109 and Y111 of SAL and Y658 and Y661 from I‐SET, disrupting their original interactions while forming a new network of hydrophobic interactions (Figure 3B). Upon H3K27me3 binding, EED allosterically stimulates the methyltransferase activity of EZH2/PRC2. Disruption of the EED‐EZH2 interactions by C36 is expected to attenuate the allosteric activation of PRC2. Therefore, the remodeling of the interacting network between EZH2 and EED by C36 underlies its inhibition of PRC2. The approved anticancer drugs tazemetostat and valemetostat belong to the class of SAM‐competitive EZH2 inhibitors. The structures of PRC2 bound to this class of inhibitors (e.g., GSK‐126 and CPI‐1205) showed that their binding pockets overlapped with that of SAH/SAM, which explains their SAM‐competitive mechanism (Figure 3C). In contrast, the C36‐binding site is adjacent to, but does not overlap with, the SAM‐binding site. Both can bind simultaneously to PRC2. In fact, C36 makes direct contact with SAH and alters its binding mode in subtle ways, possibly contributing to the reduced catalysis (Figure S6C,D).

To validate the C36‐binding site observed in our structure, we mutated the C36‐binding EZH2 residues into alanine and tested their methyltransferase activities and their inhibition by C36 in in vitro biochemical assays. The PRC2 complex containing EZH2 P132A, V657A, or Y658A, had decreased methyltransferase activities, indicating that these residues were required for catalysis (Figure 3D). PRC2 complexes with EZH2 I109A, Y111A, Y661A, and C663A mutations retained their H3K27me3 methyltransferase activities, which were no longer inhibited by C36 (Figure 3D). We next expressed Flag‐tagged EZH2 P132A, V657A, or Y658A in G401 cells, a human malignant rhabdoid tumor cell line. Consistent with the in vitro results, ectopic expression of these catalytically inactive EZH2 mutants in G401 cells reduced H3K27me3 levels (Figure 3E), suggesting that their incorporation into the endogenous PRC2 complex inhibits its activity in a dominant‐negative manner. Overexpression of EZH2 wild‐type (WT), I109A, Y111A, Y661A or C663A elevated H3K27me3 levels in the presence of C36, which were resistant to C36 inhibition to varying degrees (Figure 3E–G). Dose response curves showed that these mutations all increased the IC_50_ values of C36, with the fold change ranging from 17.4 to 86.8 (Figure 3F,G and Figure S7). These results validated the C36‐binding site in PRC2 observed in our cryo‐EM structures and identified I109, Y111, Y661 and C663 as critical residues that mediate C36 binding and inhibition.

### 2.4 C36 Specifically and Potently Inhibits EZH2/PRC2 in Cancer Cells

**FIGURE 4:**
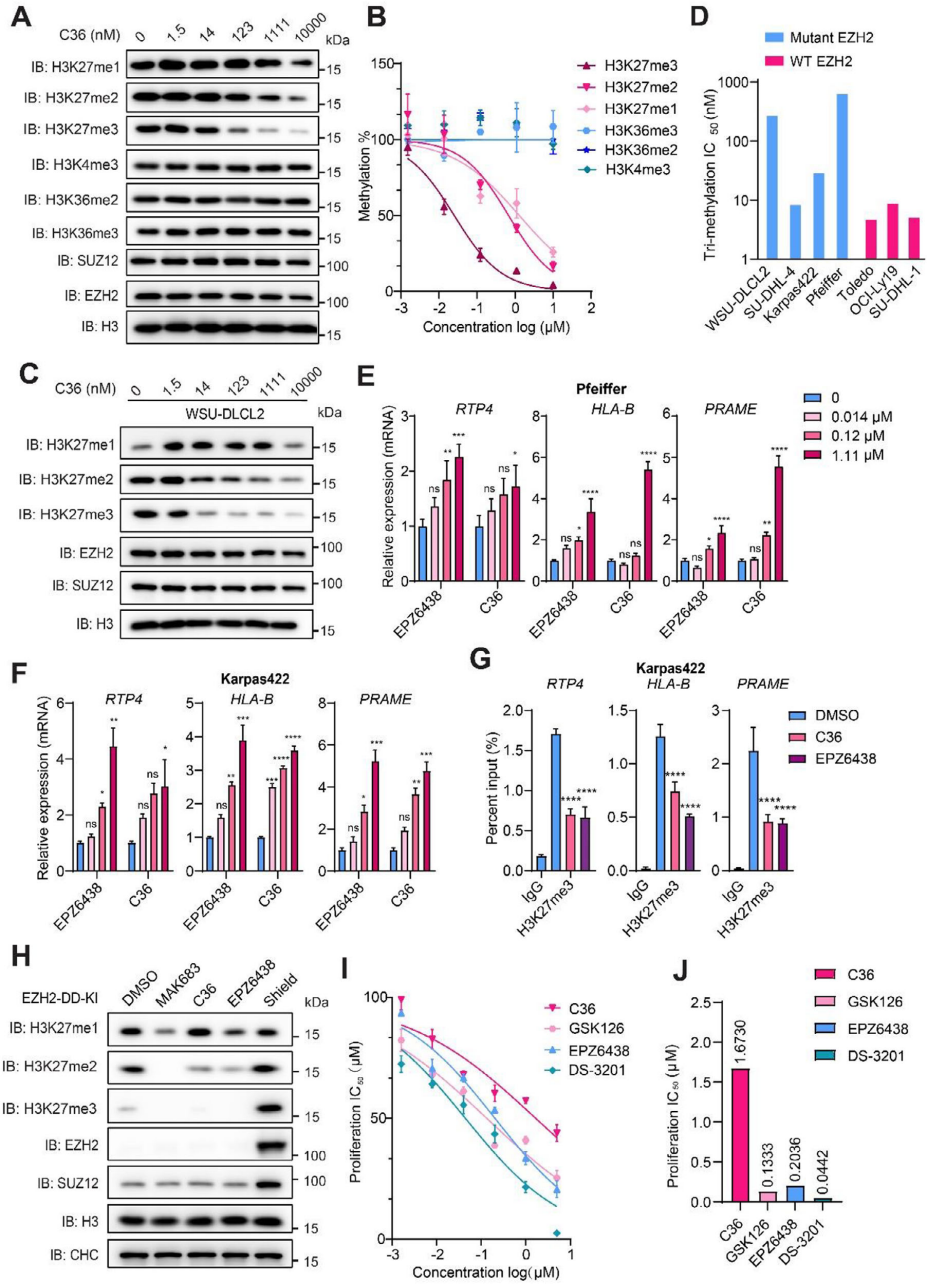
C36 inhibits PRC2 in cells. (A) Inhibition of H3 methylation by different concentrations of C36 measured by Western blot. G401 cells were treated with C36 for 3 days at the indicated concentration. Repeated 3 times. (B) Representative curves showing the inhibition of H3 methylation by increasing doses of C36 compound shown in (A) (mean ± s.d., *n* = 3). (C) Inhibition of H3K27 methylation by different concentrations of C36 measured by Western blot. WSU‐DLCL2 cells were treated with C36 for 3 days at the indicated concentration. Repeated 3times. (D) Effect of C36 on H3K27me3 marker of DLBCL cell lines with wild type EZH2 or Y641 mutated EZH2. DLBCL cell lines were treated as in (C). (E) and (F) Gene expression was determined by RT‐qPCR following 5 days treatment with C36 or EPZ6438 in Pfeiffer cells and Karpas422 cells (mean ± s.d., *n* = 3). (G) ChIP‐qPCR showed H3K27me3 at the targeted gene promoters in Karpas422 cells that were treated with DMSO, C36 (1.1 µm), EPZ6438 (1.1 µm) for 5 days. Rabbit IgG (DMSO sample) was used as a control (mean ± s.d., *n* = 4). (H) Western blot of H3K27 methylation in WSU‐DLCL2 cells. EZH2‐DD‐KI (DD‐KI) cells were withdrawn from Shield treatment for 6 days, followed by treatment with Shield (5 µm), DMSO, C36 (1.1 µm), EPZ6438 (1.1 µm), MAK683 (1.1 µm) for 4 days. Repeated 3 times. (I) Inhibition of mouse bone marrow cell proliferation was measured by the colony forming cell (CFC) assay following 12‐day treatment with the individual compounds at indicated concentrations (0.0016, 0.008, 0.04, 0.2, 1 and 5 µm) or DMSO control. (*n* = 3) (J) Effects of PRC2 inhibitors on proliferation of mouse bone marrow cell by CFC assay. The mouse bone marrow cells were treated as (I). The calculated IC_50_ values for each inhibitor are indicated above the respective bars in the graph. EPZ6438 (tazemetostat) is an FDA approved EZH2 inhibitor, as positive control. GSK126 is a highly selective EZH2 inhibitor that has been characterized in the literature. MAK683 is an allosteric EED inhibitor that lacks selectivity between EZH1 and EZH2. Statistical analysis was performed using one‐way ANOVA (^*^, *p* < 0.05; ^**^, *p* < 0.01; ^***^, *p* < 0.001; ^****^, < 0.0001).

The PRC2 complex is the only known methyltransferase for H3K27 methylation. EZH1/PRC2 catalyzes H3K27 mono‐ and di‐methylation (H3K27me1/2) whereas EZH2/PRC2 mediates the production of H3K27me1/2/3. We next assessed the cellular potency and selectivity of C36. Treatment of G401 cells with C36 led to a dose‐dependent reduction in the global levels of H3K27me3 with an IC_50_ of 25 nm (Figure 4A,B). C36 did not inhibit the production of H3K27me1/2 as efficiently, consistent with its inability to inhibit EZH1/PRC2 in vitro. The other H3 methyl marks, including H3K4me3 and H3K36me2/3 were not altered, demonstrating the selectivity of C36. Some diffused large B cell lymphoma (DLBCL) cell lines contain GOF mutations of EZH2 which altered the substrate preference of PRC2, resulting in high H3K27me3 and low H3K27me2. C36 greatly reduced H3K27me3 in DLBCL cell lines harboring EZH2 wild‐type (Toledo, OCI‐ly19, SU‐DHL‐1) or GOF mutants (WSU‐DLCL2, SU‐DHL‐4, Karpas422 Pfeiffer) (Figure 4C,D and Figure S8A). Interestingly, the SAM‐competitive PRC2 inhibitor GSK126 (IC_50_ = 70.49 nm) had an IC_50_ 5‐fold greater than that of C36 (IC_50_ = 14.16 nm) in cells (Figure S8B), despite being more potent than C36 in in vitro biochemical assays (Figure 1F,G). The high cellular SAM concentrations likely caused the reduced potency of SAM‐competitive inhibitors. Being non‐competitive with SAM, C36 thus performed better in cells.

As expected, C36 caused an upregulation of PRC2 target genes in cancer cells in a dose‐dependent manner, similar to another PRC2 inhibitor EPZ6438 (Figure 4E,F and Figure S8C,D). C36 also decreased the promoter occupancy of H3K27me3 and SUZ12 on these genes (Figure 4G and Figure S8E). C36 inhibits EZH2/PRC2 much more potently than EZH1/PRC2 in vitro (Figure 1E). We next tested whether C36 was also selective for EZH2 in human cells. EZH1 and EZH2 are mutually exclusive within PRC2 and EZH2 shows higher methyltransferase efficiency under the same reaction conditions [31]. EZH1 is dispensable and EZH2 loss results in early embryonic lethality during gastrulation in mice [32]. *Ezh2^−/−^* embryonic stem (ES) cells retained robust H3K27me1 but little H3K27me2 or H3K27me3, indicating that EZH1 contributes to H3K27me1 when EZH2 is absent [33]. We established a WSU‐DLCL2 cell line with inducible EZH2 degradation. In these cells, the endogenous EZH2 protein was N‐terminally tagged with the compound Shield‐stabilized degron [3]. Withdrawal of Shield led to the degradation of DD‐EZH2, resulting in a significant decrease of H3K27me3 without affecting H3K27me1/2 (Figure 4H). Thus, in these cells, EZH1 mediated most H3K27me1/2 modifications. The EED inhibitor MAK683 efficiently reduced EZH1‐mediated H3K27me1/2, indicating that it inhibited both EZH1 and EZH2. EPZ6438 also reduced H3K27me1/2, indicating that it inhibited EZH1 too. In the absence of Shield, C36 did not alter H3K27me1 levels and only moderately reduced H3K27me2, indicating that C36 did not efficiently inhibit EZH1 (Figure 4H). Thus, C36 is more selective in inhibiting EZH2/PRC2 than other PRC2 inhibitors in human cancer cells.

### 2.5 Assessment of EZH2/PRC2 Inhibitors Hematopoietic Toxicity Assays

A major challenge with previous SAM‐competitive inhibitors is their lack of selectivity for EZH2 over EZH1, which has been linked to mechanism‐based toxicities. For example, EPZ6438 exhibited hematopoietic toxicities such as thrombocytopenia and anemia in clinical trials [34], which are consistent with the mechanism‐based effect of disrupting both EZH1/PRC2 and EZH2/PRC2 in hematopoietic stem cells and progenitors [35, 36]. Given that C36 demonstrates an exceptional selectivity for EZH2, being over 440‐fold more potent for EZH2 inhibition than for EZH1 (Figure 1F), we compared C36 with other EZH2/PRC2 inhibitors in a hematopoietic colony‐forming cell (CFC) assay. Mononuclear cells harvested from mouse bone marrow were cultured in a semi‐solid methylcellulose medium with different PRC2 inhibitors for 12 days. C36 exhibited significantly lower toxicity to bone marrow cells than GSK126 (by 13‐fold) (Figure 4I,J). Similarly, C36 was 8‐fold and 40‐fold less toxic than EPZ6438 (tazemetostat) and DS‐3201 (valemetostat), respectively (Figure 4I,J). We also assessed short‐term hematological toxicity using a CCK‐8 assay. Mouse bone marrow cells were treated with the indicated PRC2 inhibitors for 6 days. C36 again demonstrated the lowest cytotoxicity compared to the others (Figure S8F,G). So, C36 is less toxic compared to other EZH2/PRC2 inhibitors in mouse bone marrow.

### 2.6 C36 Inhibits Cell Proliferation and Xenograft Tumor Growth

**FIGURE 5:**
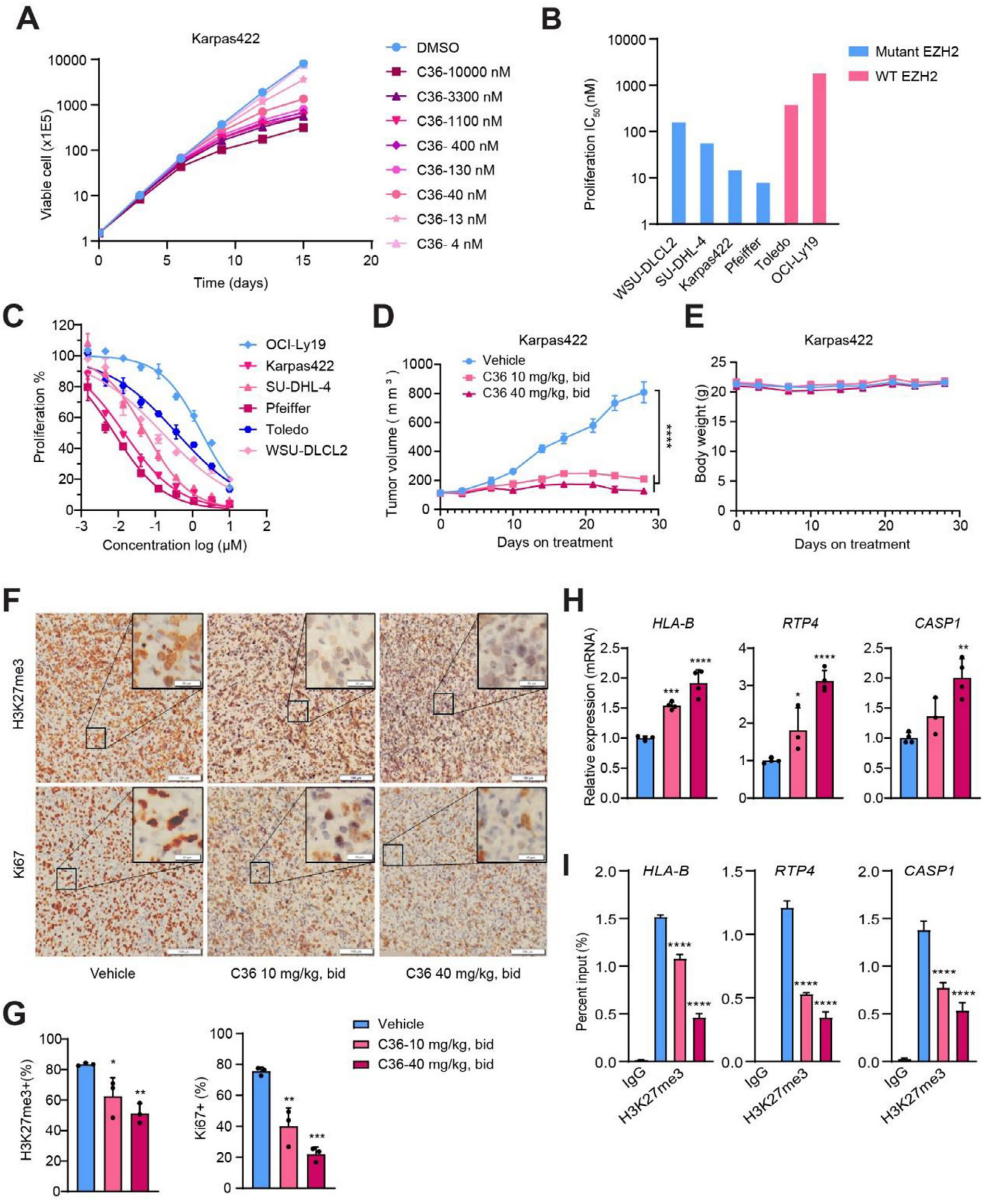
C36 inhibits lymphoma proliferation and regresses tumor in the xenograft model. (A) Dose‐dependent effects of C36 on cell proliferation over time in Karpas422 cells (mean ± s.d., *n* = 2). (B) Effect of C36 on proliferation of DLBCL cell lines with wild type EZH2 or Y641 mutated EZH2. DLBCL cell lines were treated as in (A) at day 12, Pfeiffer at day 9. Proliferation of IC50 means the concentration of C36 required to inhibit 50% growth. (C) Representative curves showing the inhibition of DLBCL cell proliferation by increasing doses of C36 compound shown in (A) (mean ± s.d., *n* = 2). (D) Growth curve of subcutaneous Karpas422 xenograft tumors in mice treated with C36 (10 mg kg^−1^, 40 mg kg^−1^) or vehicle orally twice a day (mean ± s.e.m., *n* = 5). (E) Body weight of the mice carrying the subcutaneous Karpas422 xenografts treatment as in (D) (mean ± s.e.m., *n* = 5). (F) Representative H3K27me3 and Ki67 IHC images of tumor samples from the end point of study in (D). Scale bar for images, 100 µm; scale bar for the intersects, 20 µm. (G) The percentage of H3K27me3 and Ki67 positive cells in tumor samples (mean ± s.d., *n* = 3 mice per group). (H) Gene expression was determined by RT‐qPCR in tumor samples from (D) (mean ± s.d., *n* = 4 mice per group). (I) ChIP‐qPCR showed H3K27me3 at the targeted gene promoters in tumor samples from (D). Rabbit IgG (Vehicle sample) was used as a control (mean ± s.d., *n* = 4). Statistical analysis was performed using one‐way ANOVA (^*^, *p* < 0.05; ^**^, *p* < 0.01; ^***^, *p* < 0.001; ^****^, < 0.0001).

Lymphoma cell lines carrying EZH2 GOF mutations are sensitive to PRC2 inhibitors [9, 37, 38]. C36 inhibited the proliferation of Karpas422 cell carrying the EZH2‐Y641N mutation (Figure 5A). The multiple lymphoma cell lines with EZH2 GOF mutations were indeed more sensitive to C36 (Figure 5B,C). Next, C36 was evaluated in mice using subcutaneous xenografts of Karpas422. We dosed C36 via the oral route (p.o.) twice daily at 10 and 40 mg/kg and observed significant tumor growth inhibition after 10 days and mild tumor regression after 20 days (Figure 5D). C36 at each dose was well tolerated in animals, as indicated by no drop of body weight and the absence of obvious adverse effects, such as hypothermia or reduced activities (Figure 5E). Immunohistochemistry (IHC) results confirmed a dose‐dependent decrease of H3K27me3 and the proliferation marker Ki67 in tumor tissues (Figure 5F,G). The representative PRC2 target genes were also upregulated in a dose‐dependent manner (Figure 5H). ChIP‐qPCR results confirmed that the promoter H3K27me3 and SUZ12 levels of these genes were decreased in tumor samples with C36 dosing (Figure 5I and Figure S9A). Thus, C36 demonstrates a dose‐dependent PD modulation and efficacy in mouse tumor xenografts in vivo.

### 2.7 C36 Treatment Triggers Interferon Signaling

**FIGURE 6:**
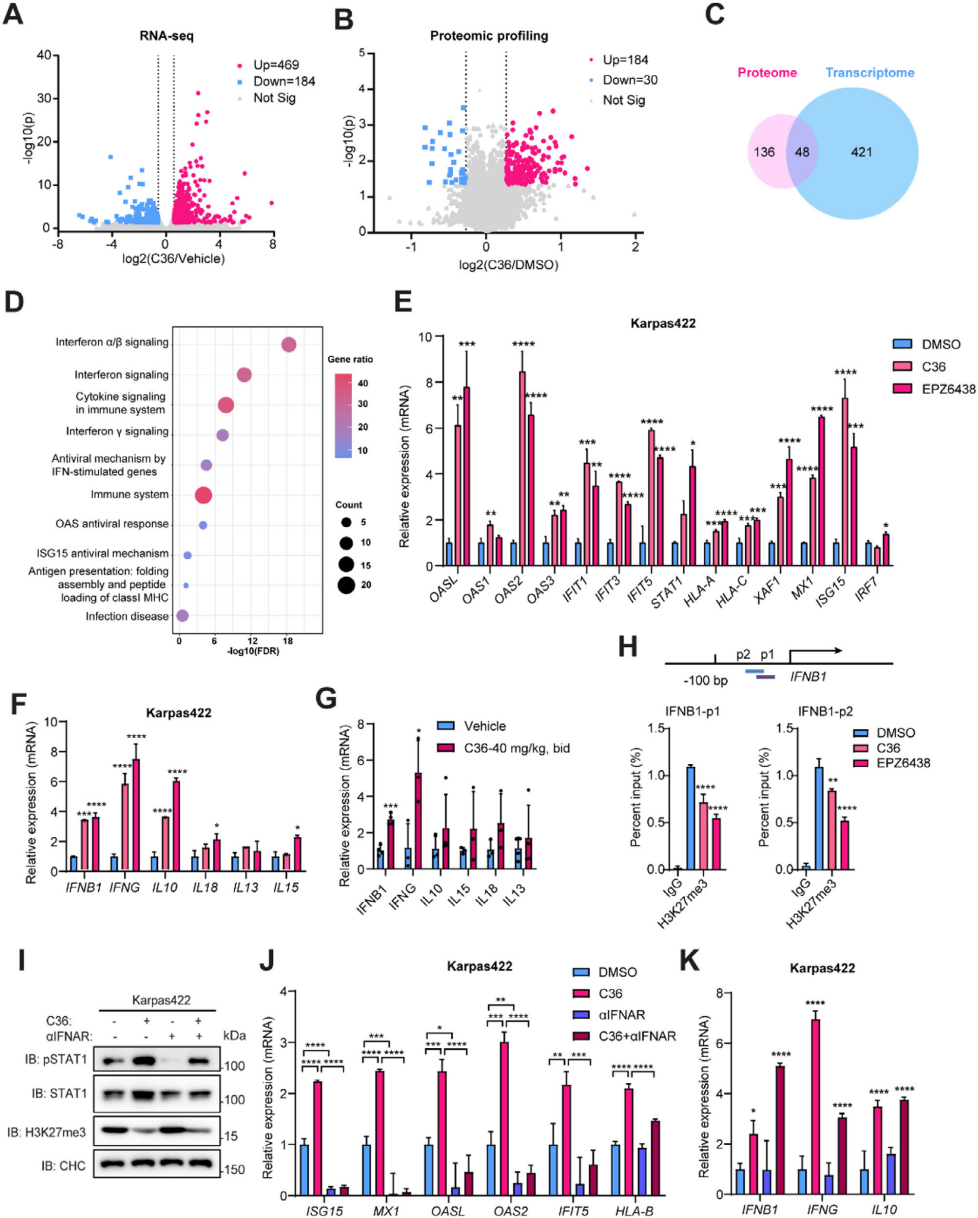
C36 treatment triggers interferon signaling. (A) Volcano plots showing the differentially expressed genes (DEGs) in xenograft tumors with dosing of C36 (40 mg kg^−1^) or vehicle in Figure 5D (*n* = 3 independent tumors). Red indicates upregulation with C36 (fold change ≥ 1.5 and *p* adjust ≤ 0.05) and blue indicates downregulation with C36 (fold change ≤−1.5 and *p* adjust ≤ 0.05). (B) Volcano plots showing the differentially expressed proteins in Karpas422 cells with treatment of C36 or DMSO. Red indicates upregulation with C36 (fold change ≥ 1.2 and *p* adjust ≤ 0.05) and blue indicates downregulation with C36 (fold change ≤−1.2 and *p* adjust ≤ 0.05). (C) Venn diagram of upregulated genes in (A) and upregulated proteins in (B). (D) The bubble plot with changed biological processes assigned according to reactome classification. The bubble size indicates the number of genes represented in the corresponding annotation. The color of each bubble reflects the gene ratio. (E) Gene expression associated with the interferon signaling pathway, as shown in (D), was determined by RT‐qPCR. Karpas422 cells were treated with DMSO, C36 (1.1 µm), EPZ6438(1.1 µm) for 5 days (mean ± s.d., *n* = 3). (F) Gene expression associated with biocarta—cytokine pathway (Figure S5E,F) was determined by by RT‐qPCR. Karpas422 cells were treated with DMSO, C36 (1.1 µm), EPZ6438 (1.1 µm) for 5 days (mean ± s.d., *n* = 3). (G) Gene expression associated with biocarta—cytokine pathway was determined by by RT‐qPCR. Tumor samples from the end point of study in Figure 5D (mean ± s.d., *n* = 4 mice per group). (H) ChIP‐qPCR showed H3K27me3 at the *IFNB*1 promoter in Karpas422 cells that treated with DMSO, C36 (1.1 µm), EPZ6438(1.1 µm) for 5 days. Rabbit IgG (Vehicle sample) was used as control (mean ± s.d., *n* = 3). (I) Western blots of pSTAT1 or the indicated proteins in Karpas422 cells treated with C36 (1.1 µm), and αIFNAR1 (Anifrolumab, 10 µg/mL) for 3 days. Repeated 3 times. (J) and (K) IFN‐stimulated genes (ISGs), and cytokine genes expression were determined by RT‐qPCR. Karpas422 cells were treated with DMSO, C36 (1.1 µm), αIFNAR1 (Anifrolumab, 10 µg/mL) for 5 days (mean ± s.d., *n* = 4). Statistical analysis was performed using one‐way ANOVA (*E*, *F*, *H*, *J*, *K*) or two‐tailed unpaired t‐test (G) (^*^, *p* < 0.05; ^**^, *p* < 0.01; ^***^, *p* < 0.001; ^****^, < 0.0001).

To better understand the effects of PRC2 inhibition by C36 in tumors, we performed RNA‐seq using the tumor samples from the endpoint of the study in Figure 5D. C36 dosing resulted in the significant upregulation of 469 genes and downregulation of 184 genes in tumors by Differentially Expressed Genes (DEGs) analysis (Figure 6A and Figure S9B). Pathways related to innate immunity, particularly the type I interferon pathway, were highly enriched in the C36 group (Figure S9C). We also performed mass spectrometry‐based quantitative proteomics using the Tandem Mass Tag (TMT) analysis. We identified 214 differentially expressed proteins (DEPs) in C36‐treated Karpas422 cells, among which 184 were upregulated while 30 were downregulated (Figure 6B). The upregulated DEGs and DEPs shared 48 genes in common (Figure 6C). Enrichment analysis of these 48 genes indicated interferon (IFN) signaling was still the predominant upregulated pathway upon C36 treatment (Figure 6D). The top enriched genes included Major histocompatibility complex (MHC) class I molecules (HLA‐A/B/C), oligoadenylate synthases family (OAS1/2/3) and IFIT family members (IFIT1/3/5), which were all associated with innate immunity and induced by interferon signaling (Figure S9D). The upregulation of these genes was further validated by RT‐qPCR in both cells treated with EPZ6438 or C36 and xenograft tumor samples (Figure 6E and Figure S9E). In our previous study, we also observed that interferon signaling was significantly upregulated in WSU‐DLCL2 and patient‐derived xenograft tumors following MAK683 treatment [3]. Thus, C36 inhibits PRC2 in tumors and induces interferon signaling, like other PRC2 inhibitors.

Recent studies have reported that PRC2 can negatively regulate type I interferon (IFN) signaling by directly silent MHC class I genes in most cell types [2] and type I IFN ligands in ERα+ breast cancer cells [39]. In the context of DLBCL, what mechanisms are involved in the activation of interferon signaling upon PRC2 inhibition? We found that *IFNB1* and *IFNG* were upregulated by C36 treatment in both Karpas422 cells and tumor xenografts (Figure 6F,G and Figure S9F,G). ChIP‐qPCR showed a reduction of H3K27me3 at the *IFNB1* promoter region following C36 treatment, consistent with its de‐repression (Figure 6H). Furthermore, C36 activated STAT1 phosphorylation and the upregulation of interferon‐stimulated genes (ISGs) (Figure 6I,J). This upregulation was blunted by a neutralizing antibody against IFNAR1 (αIFNAR), which blocked IFN signaling. The upregulation of *HLA‐B* and *IFNG* by C36 was partially affected by αIFNAR while the upregulation of *IFNB1* and *IL10* was not affected (Figure 6J,K). These results suggest that EZH2/PRC2 inhibition by C36 directly activates the expression of *IFNB1* in DLBCL, which in turn stimulates interferon signaling and associated downstream events.

### 2.8 Combined Treatment With C36 and Immunotherapeutic Strategies Improves Anti‐Tumor Efficacy

**FIGURE 7:**
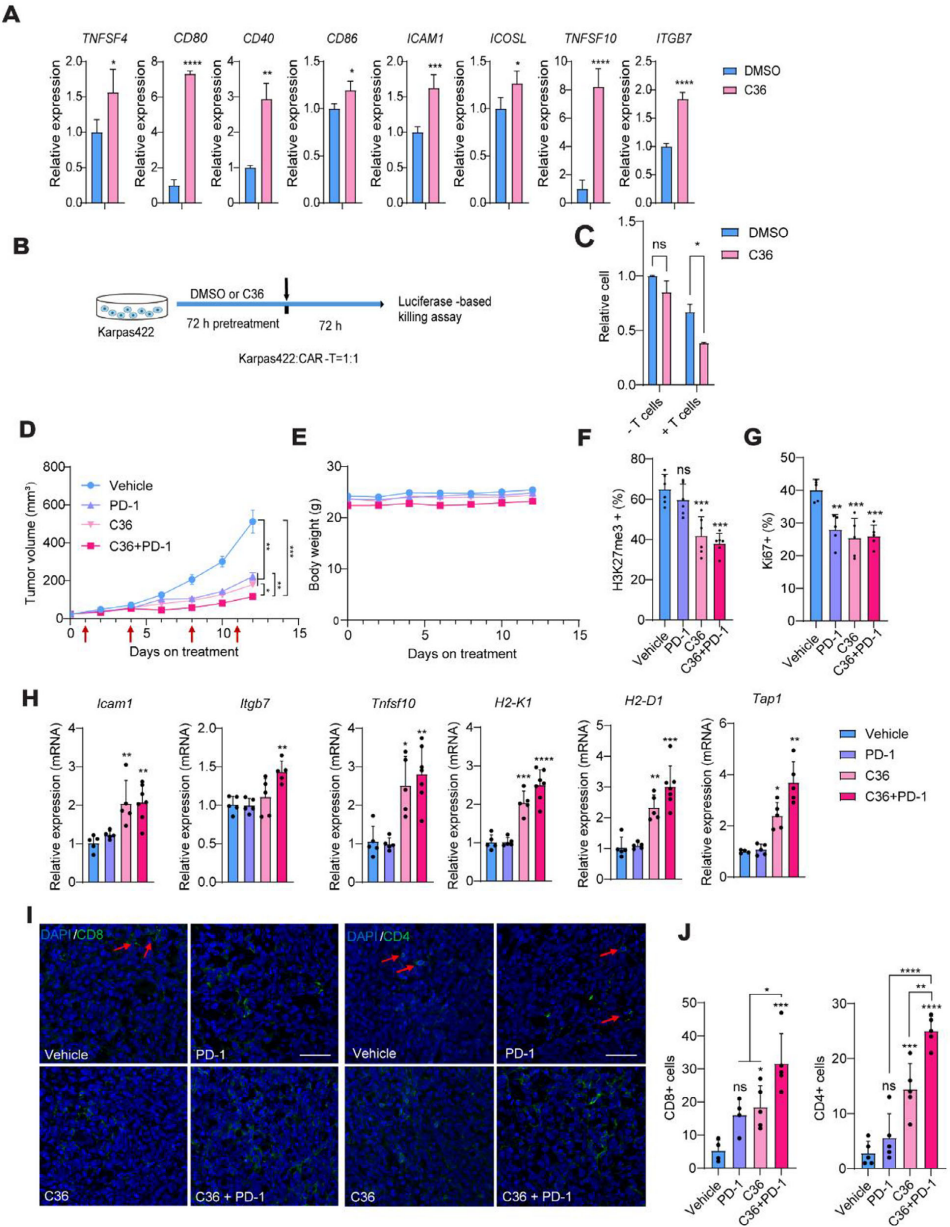
Combined treatment with C36 and immunotherapeutic strategies improves anti‐tumor efficacy (A) Gene expression was determined by RT‐qPCR. Karpas422 cells were treated with DMSO or C36 (1.1 µm) for 5 days (mean ± s.d., *n* = 4). (B) Procedure of C36 and CART19 administration. (C) In the cell killing assay, Karpas422‐luc cells were pretreated for 3 days with 20 nm of C36 or DMSO, and then co‐cultured with or without CART19 (*n* = 2). Repeated 3 times with biological repeats. (D) Growth curve of subcutaneous LLC tumors in C57Bl/6 mice treated with twice‐daily oral C36 (30 mg kg^−1^) or vehicle and subcutaneous anti‐PD‐1 (10 mg kg^−1^) or PBS. Red arrows indicate time points for anti‐PD‐1 antibody administration (mean ± s.e.m., *n* = 9). (E) Body weight of the mice carrying the subcutaneous LLC tumors treatment as in (D) (mean ± s.e.m., *n* = 9). (F) and (G). The percentage of H3K27me3 and Ki67 positive cells in tumor samples (mean ± s.d., *n* = 5 mice per group). (H) Gene expression was determined by RT‐qPCR, tumor samples from the end point of study in (D) (mean ± s.d., *n*≥5). (I) Representative immunofluorescence (IF) images of CD8^+^ and CD4^+^ T cell infiltration in tumor samples collected at the end point of study in (D). Scale bar for images, 50 µm. (J) The percentage of CD8 and CD4 positive cells in tumor samples (mean ± s.d., *n*≥4 mice per group). Statistical analysis was performed using one‐way ANOVA (F,G,H,J), two‐tailed unpaired t‐test (A), two‐way ANOVA (C) and Brown‐Forsythe and Welch ANOVA tests (D) (^*^, *p* < 0.05; ^**^, *p* < 0.01; ^***^, *p* < 0.001; ^****^, < 0.0001).

The IFN signaling plays a critical role in tumor immunotherapy [40, 41, 42]. To investigate whether PRC2 inhibition by C36 enhances tumor responsiveness to immune checkpoint inhibitors, we treated Karpas422 cells with the C36 and observed the upregulation of multiple immunomodulatory genes, including adhesion molecules and integrins (e.g., *ITGB7*) (Figure 7A), co‐stimulatory receptors (*CD86*, *CD80*, and *CD40*) (Figure 7A), MHC class I genes (*HLA‐A*, *HLA‐B*, and *HLA‐C*) (*HLA‐B* in Figure 5H, *HLA‐A* and *HLA‐C* in Figure 6E), as well as ligands involved in immune synapse formation with T cells (*TNFSF10*, *TNFSF4*, *ICOSL*, and *ICAM1*)(Figure 7A). These findings indicate that PRC2 inhibitors remodel tumor cells, potentially priming a response to immunotherapies. To assess the functional consequence of this remodeling, we co‐cultured Karpas422 cells, pretreated with either C36 or DMSO, with anti‐CD19 CAR‐T cells (Figure 7B). Using stably transfected luciferase as a measure of viable Karpas422 lymphoma cells, we observed that C36 pretreatment significantly enhanced the elimination of tumor cells by CAR‐T cells (Figure 7C).

We further validated these findings in an immune‐competent in vivo model by transplanting mouse lung cancer LLC cells into C57BL/6 mice. While both C36 and an anti‐PD‐1 antibody demonstrated single‐agent anti‐tumor activity, combination of them resulted in a significantly enhanced therapeutic effect without extensive toxicity (Figure 7D,E and Figure S11A). PRC2 inhibition in vivo was confirmed by reduced H3K27me3 levels and was associated with transcriptional reprogramming, including the upregulation of MHC class I and II molecules, T cell synapse ligands, and type I IFN‐related genes (Figure 7F,H and Figure S11B,D). Accordingly, proliferation marker Ki67 was reduced in all treatment groups (Figure 7G and Figure S11C). Analysis of the CD4 and CD8 in tumor showed that PRC2 inhibition promoted infiltration of CD4+ and CD8+ T cells, which was most pronounced in the combination treatment group (Figure 7I,J). Together, our results demonstrate that combining PRC2 inhibitors with immunotherapy significantly enhances anti‐tumor efficacy by reprogramming cancer cells and promoting a more robust anti‐tumor immune response.

## 3 Discussion and Conclusions

Dysregulated epigenetic state is a hallmark of cancer and often arise from genetic alterations in epigenetic regulators, such as the multi‐component methyltransferase PRC2. Tazemetostat and valemetostat are the only two clinically approved inhibitors for treating PRC2‐related cancers. Other inhibitors, including EED binders and PROTAC degraders, are at various stages of development. Moreover, combining EZH2 inhibitors with other therapeutic approaches, such as chemotherapy and immunotherapy, has been shown to enhance the therapeutic efficacy and overcome drug resistance even in the context of immunosuppressive tumors. Thus, there are still needs to develop novel PRC2 inhibitors.

Here we have characterized C36, a PRC2 inhibitor with a unique mechanism of action. Being non‐competitive with SAM, C36 binds to a site on PRC2 that does not overlap with the SAM‐binding site. C36 binding remodels the interaction network between EZH2 and ligand‐bound EED, thus disrupting the allosteric activation of EZH2 by EED. C36 also directly contacts SAM, altering its binding to EZH2 in subtle ways and possibly perturbing catalysis. The direct interaction between C36 and SAM/SAH stabilizes SAM binding to EZH2 and reduces its Km. In cancer cells which have high cellular SAM concentrations, C36 is thus expected to be more effective in PRC2 inhibition as compared to SAM‐competitive inhibitors.

We show that several C36‐binding residues, including P132 from SRM and V657 and Y658 from I‐SET, are critical for the catalytic activity of PRC2 (P132 has previously been reported to be critical for the activity of PRC2 [43]).Binding of C36 to these residues alters the interactions among these functionally important residues, thus inhibiting PRC2. Mutations of several other C36‐binding residues do not abolish the activity of PRC2 but blunt the inhibition by C36. While validating the C36‐binding site identified by our structural analysis, these results also reveal potential mechanisms of drug resistance. Indeed, mutations of residues in this pocket, including I109, Y111 and Y661, contribute to acquired resistance to SAM‐competitive inhibitors, such as EPZ6438 [44, 45]. These mutants will likely also confer resistance to C36 inhibition and can be overcome by EED binders. These findings highlight the need for the development of PRC2 inhibitors with different mechanisms of action.

C36 exhibits high selectivity for EZH2/PRC2 over EZH1/PRC2. C663 of EZH2 is critical for C36 binding, and the equivalent residue in EZH1 is S664 (Figure S10). This cysteine‐to‐serine substitution likely contributes to the resistance of EZH1 to C36 inhibition. In addition, EZH1/PRC2 is not stimulated by H3K27me3 binding to EED, likely due to the replacement of H129 in EZH2 by C130 in SRM of EZH1 [46]. Indeed, replacing H129 with cysteine may indirectly affect the interaction between P132 and C36. Experiments with EZH1/2 chimeras are needed to further dissect the selectivity of C36 toward EZH2.

Consistent with EZH2/PRC2 being the major tumor driver in DLBCL, C36 is highly effective in reducing cellular H3K27me2/3, upregulating PRC2 target genes, and slow the proliferation of cancer cells in vitro and in xenograft models. Given its non‐competitive SAM binding and high selectivity for EZH2, C36 is anticipated to have an improved safety profile in vivo. Indeed, C36 demonstrated lower cytotoxicity in bone marrow CFC assays comparing with other EZH2/PRC2 inhibitors at the same concentration (Figure 4I,J). This marked reduction in hematotoxicity supports that C36 might show a favorable clinical safety profile.

An earlier report showed that PRC2 represses type I IFN signaling in breast cancers [39]. We have extended this finding to B cell lymphoma. We have separated direct PRC2 target genes from indirect downstream targets of IFN signaling by using an IFNAR1 neutralizing antibody. Using this antibody and C36 treatment, results showed that *HLA‐B*, *IFNB1*, and *IL10* are direct targets of PRC2 in B cell lymphoma, while other ISGs are activated by IFN signaling (Figure 6I–K). Our results have thus defined the functional outputs of PRC2 inhibition in lymphoma. Furthermore, consistent with prior research [47, 48, 49], our results also demonstrate that combining PRC2 inhibitors with immunotherapeutic strategies significantly enhances antitumor efficacy. These findings provide a compelling rationale for the combined use of PRC2 inhibitors and immunotherapy in the treatment of B‐cell lymphoma or other tumors.

In summary, we have presented compelling evidence to demonstrate that C36 is a potent and selective EZH2/PRC2 inhibitor. It inhibits EZH2/PRC2 by disrupting the allosteric communication between EZH2 and EED in a SAM‐noncompetitive manner. With high EZH2 selectivity, C36 exhibits low cytotoxicity in mouse bone marrow CFC assays. We further show that inhibition of EZH2/PRC2, but not EZH1/PRC2, by C36 suffices to block tumor growth in mouse xenograft models in part by inducing interferon signaling. Combined treatment of a syngeneic model with C36 and PD‐1 antibody improves anti‐tumor efficacy. Our study lays the foundation for the design and development of next‐generation EZH2/PRC2 inhibitors for cancer therapy.

## 4 Materials and Methods

### 4.1 Protein Expression and Purification

The PRC2‐related proteins were purified following a modified method described by Eva [50]. The cDNA of EZH2 was cloned from the H9 human embryonic stem cell line, while others were synthesized by Genscript. The unstructured regions of SUZ12 were truncated to obtain a 71–685aa construct. Additionally, a minimal construct of JARID2 (1–450aa) was created. All proteins were cloned into the pFastBac or pLIB vector using an N‐terminal Twin‐strep tag and His6 tag on AEBP2, or an N‐terminal 3*FLAG tag on JARID2 (aa 1–450), each with a TEV cleavage site after the affinity tag. Furthermore, EZH2 and EED were tagged directly before the start codon with an N‐terminal His8 affinity tag. All constructs were expressed in High Five cells using the Bac‐to‐Bac baculovirus system (Invitrogen). The cells were infected with P2 Baculovirus and incubated at 27°C for 50–60 h before harvesting for protein extraction and purification. For protein extraction, High Five cell pellets infected with PRC2 complex‐containing virus were lysed in Buffer A (25 mm HEPES pH 7.5, 250 mm NaCl, 2 mm MgCl_2_, 1 mm TCEP, 0.1% NP40, 5% glycerol, and 0.2 mm PMSF). All purification steps were carried out at 4°C. The cells were lysed by sonication or an UltraHigh Pressure Homogenizer and then centrifuged twice at 16 000 rpm for 25 min at 4°C. The supernatant was incubated with Strep‐Tactin superflow resin (Qiagen) or anti‐FLAG M2 affinity agarose for 1–2 h. Subsequently, the resin was washed with 20 column volumes (CV) of wash buffer B (25 mm HEPES pH 7.5, 250 mm NaCl, 2 mm MgCl_2_, 0.01% NP40, 1 mm TCEP, 5% glycerol) and then washed with 5 CVs of elution buffer without desthiobiotin (25 mm HEPES pH 7.5, 250 mm NaCl, 2 mm MgCl_2_, 1 mm TCEP, 5% glycerol). The protein was eluted with 10 mm desthiobiotin or 150 µg/ml 3xFLAG peptide in elution buffer, and incubated with HRV 3C protease overnight at 4°C. Following elution, the protein was purified using size‐exclusion chromatography with a Superose 6 increase 3.2/300 column (GE Healthcare) in a running Buffer A (25 mm HEPES pH 7.5, 150 mm NaCl, 2 mm MgCl_2_, 1 mm TCEP, and 5% glycerol). The peak fractions were collected for biochemical assays, electron microscopy experiments, or frozen using liquid nitrogen and stored long‐term at −80°C.

### 4.2 PRC2 Biochemical Assay by LC/MS

The IC_50_ value and Michaelis–Menten equation were determined using liquid chromatography mass spectrometry (SCIEX QTRAP 6500+), following the reported method. To determine the IC_50_, compounds were serially diluted three‐fold in dimethyl sulfoxide (DMSO) to obtain a total of eight or twelve concentrations. These dilutions were then further diluted in the assay buffer to reach the final concentrations. The typical reaction (8 µL) consisted of 2 µm S‐adenosylmethionine (SAM) at 10*Km, 50 µm H3K27me0 peptide at 10*Km, and 40 nm PRC2 in an assay buffer composed of 20 mm Tris‐HCl pH 7.4, 0.01% Triton X‐100, 0.5 mm dithiothreitol (DTT), and 0.04% bovine serum albumin (BSA). For Michaelis–Menten equation, SAM or H3K27me0 peptides were serially diluted into the indicated concentrations. The initial velocities (V_0_) of enzyme activity were determined by calculating the slopes of indicated time points of each concentration.

The reactions were stopped by adding 24 µL methanol and thoroughly mixing. Subsequently, 30 µL of water containing 200 nm SAH‐d4 (Cayman chemical) was added to achieve a final concentration of 100 nm. Liquid chromatography was carried out using an ACQUITY UPLC HSS T3 Column (1.8 µm, 2.1 mm x 50 mm, Waters). The column was directly connected to the turbo ion electrospray and operated in positive‐ion mode. The m/z values for the parent ions of SAH and SAH‐d4 are 385.2 and 389.3, respectively, while the m/z values for the daughter ions of SAH and SAH‐d4 are both 136.1. The mobile phase A consisted of 0.1% formic acid (FA) and 10 mm ammonium formate in water, and mobile phase B consisted of 0.1% FA in acetonitrile. An injection volume of 2 µL was used, and the autosampler was maintained at 4°C. The flow rate was set at 1 mL/min. Eluents from 1–5 min were directed to the mass spectrometer for analysis. The peaks corresponding to SAH and SAH‐d4 appeared at approximately 2.3 min, and the ratio of SAH peak area to SAH‐d4 peak area, as a function of SAH concentration, was plotted to generate the normalization factor for SAH. The production of SAH from the actual enzymatic reaction was obtained from the standard curve of SAH. The data were analyzed using GraphPad Prism.

### 4.3 AlphaLISA Immunoassays

The AlphaLISA assay was conducted in ChemPartner. Briefly, it was performed utilizing the AlphaLISA EZH2 Histone H3‐Lysine 27 N‐methyltransferase assay. C36 and GSK126 were serially diluted (10 concentrations, down‐titration from 10 µM with a 1:3 dilution factor) in 384‐well plates. Five microliters of a compound or DMSO were transferred to the assay plate, with a final DMSO concentration of 1%. Transfer 5 µL of enzyme solution (EZH1/EZH2, EED, SUZ12, RBBP4, AEBP2 in 1x Epigenetics Buffer) to the assay plate or for low control transfer 5 µL of 1x assay buffer. The plate was then incubated at room temperature for 15 min. Next, add 5 µL of substrate solution to each well to start the reaction, followed by incubation at room temperature for 60 min. Add 15 µL beads mix solution, incubate for 60 min at RT, subdued light. Reading endpoint with EnVision. Fit the data in Excel to obtain inhibition values using Equation: Inh % = (Max‐Signal)/ (Max‐Min) *100.

### 4.4 Histone Methyltransferase Assays by WB

HMTase assays on substrates were performed with the concentrations of PRC2 and substrates indicated in the figures, in the assay buffer containing 20 µm SAM in a total volume of 20 µl. Reactions were incubated for 45 min at 25°C and stopped by the addition of 5 µL 5× SDS gel‐loading buffer. Proteins were separated on 4%–20% Bis‐Tris MES polyacrylamide gels, and antibodies for western blots were: anti‐H3K27me3 (1:1,000), anti‐H3 (1:2000), anti‐His‐tag (1:3000), anti‐Flag (1:5000).

### 4.5 Cryo‐EM Data Collection and Image Processing

Following a modified method described by Eva [50], 6.7 µm PRC2 complex was incubated with 134 µm C36 molecule alone or 67 µm SAH and H3K27me3 or all of them for 15 min at room temperature. Then cross‐linker BS3 was added to a final concentration of 0.5 mm for 45 min, and the buffer was exchanged into 25 mm HEPES pH 7.9, 150 mm NaCl, 1 mm TCEP, 2 mm MgCl_2_. Then samples were changed and diluted to 0.3 or 0.6 mg/mL in buffer 25 mm HEPES pH 7.9, 50 mm NaCl, 1 mm TCEP, 0.01% NP‐40. For cryo‐EM grid preparation, 3 µL samples were applied onto glow‐discharged holey carbon grids with 2 nm carbon film (Quantifoil Cu R1.2/1.3, 300 mesh), blotted with a Vitrobot Marker IV (Thermo Fisher Scientific) for 3 s under 100% humidity at 4°C, and subjected to plunge freezing into liquid ethane. All cryo‐EM data were collected using the FEI Titan Krios microscope at 300 kV equipped with a Gatan K3 Summit direct electron detector (super‐resolution mode, at a nominal magnification of 81 000) and a GIF‐quantum energy filter. Defocus values were set from −1.0 to −2.0 µm. Each stack of 32 frames was exposed for 2.13 s, with a total electron dose of 50 e^−^/Å^2^. AutoEMation was used for fully automated data collection [51].

All micrograph stacks were motion‐corrected with MotionCor2 [52] with a binning factor of 2, resulting in a pixel size of 1.0773 Å. Contrast transfer function (CTF) parameters were estimated using Gctf [53]. Image processing was performed using cryoSPARC [54]. For 3D processing of the PRC2(5)‐C36, PRC2(5)‐H3K27me3‐SAH, PRC2(5)‐H3K27me3‐SAH‐C36 data, a total of 2,133,628, 2,716,189, and 2,927,647 particles were automatically picked from micrographs using Gautomatch or blob_picker of cryoSPARC. Particles were extracted with a pixel size of 4.31 Å or 440 pixels and subjected to several rounds of reference‐free 2D classification. Then, the generated ab initio models or an imported volume of 6C23 or 6C24 were used for heterogeneous 3D refinement. The best class of 239,050, 215,064, and 345,268 particles, respectively, were reextracted without binning. After the last round of 3D classification, 67,922, 82,581, and 172,729 particles were used for further 3D refinement, including homologous refinement, heterogeneous refinement, non‐uniform refinement, and local refinement. The global resolution of the PRC2 is 3.59, 3.07, 2.82 Å based on the Fourier Shell Correlation (FSC) 0.143 criterion (Figures S3–S5).

### 4.6 Model Building and Refinement

The x‐ray coordinates of human PRC2 (PDB: 5WG6 and 5HYN) were used as the starting models and docked into the final EM maps with UCSF Chimera [55]. The Alphafold3 model for SUZ12 was docked into the EM maps. The models were manually adjusted and iteratively built in COOT [56] and then refined against summed maps using phenix.real_space_refine implemented in PHENIX [57] until the validation data were reasonable. FSC values were calculated between the resulting models and the two half‐maps, as well as the averaged map of the two half‐maps. The quality of the models was evaluated with MolProbity [58] and EMRinger [59]. The structure validation statistics were listed in Table. EV1. All structural figures were prepared with PyMOL [60], Chimera [55] or Chimera X [61].

### 4.7 Cell Culture and Reagents

Cells were obtained from ATCC and maintained in a humidified incubator at 37°C with 5% (v/v) CO2. G401 was cultured in DMEM (Invitrogen, 12100046) with 10% (vol/vol) FBS (Lonsera, S711‐001S), 0.055 mm 2‐mercaptoethanol (Sigma, M3148), 100 U ml^−1^ penicillin and 100 µg mL^−1^ streptomycin. Lymphoma cells were cultured in RPMI1640 (Invitrogen) with 15% FBS, 100 U mL^−1^ penicillin and 100 µg mL^−1^ streptomycin. All cells are authenticated by STR profiling and confirmed of mycoplasma‐free. Chemical compounds EPZ6438, MAK683, and C36 were purchased from MCE, Selleck, and LinkChem.

### 4.8 Mouse Xenograft Studies

The animal study design and performance were evaluated and approved by the Institutional Animal Care and Use Committee of ShanghaiTech University under the document number of 20201225001 and following the internationally recognized guidelines on animal welfare. To establish xenograft models, Karpas422 cells were washed and resuspended in cold PBS, mixed with Matrigel at 1:1 (v/v) to reach 1 × 10^7^ cells per 200 µL, and then the tumor cell mixtures were injected subcutaneously into the left flank of female Balb/c nude mice at 200 µL per mice (5–6 weeks of age). To establish a syngeneic model, LLC cells were washed and resuspended in cold PBS (4 × 10^5^cells per 200 µL), and then the tumor cell mixtures were injected subcutaneously into the left flank of male C57BL/6 mice. When tumor volume reached ∼150 mm^3^, the mice were numbered and random‐ grouped into vehicle, 10 and 40 mg/kg C36 treatment groups. For LLC model, when tumor volume reached 20 mm^3^, the mice were numbered and random‐ grouped into vehicle, 30 mg/kg C36, 10 mg/kg anti‐PD‐1 antibody (MCE, HY‐P99144) and combination (30 mg/kg C36 and 10 mg/kg anti‐PD‐1 antibody) treatment groups.The mice with tumor size out of the range were not included in the study. Empirically, at least 5 mice were included for each group. For compound daily dosing, C36 was resolved and suspended in water with 0.5% HPMC and 0.5% Tween‐20, anti‐PD‐1 antibody was resolved in PBS. Mice weight and tumor volumes are measured and recorded twice a week. Tumor volumes were calculated based on perpendicular length and width caliper measurements using the following formula: tumor volume (mm^3^) = 0.5 × (length × width^2^).

### 4.9 Immunohistochemistry

For immunohistochemistry staining, freshly isolated tumor tissues were fixed in 4% paraformaldehyde for 24 h at room temperature. Tissues were embedded in paraffin, sectioned at thicknesses of 5 µm, deparaffinized and rehydrated through graded concentrations of ethanol in water. For antigen retrieval, sections were boiled in a microwave for 15 min in 10 mm citrate buffer (pH 6.0) for Ki67 (Abcam, ab16667; dilution 1:100), H3K27me3 (CST, 9733; dilution 1:200). After cooling down to room temperature, endogenous peroxidase was inactivated with 3% H_2_O_2_ in methanol. Then the sections were washed with dH_2_O twice, blocked with 5% normal goat serum in PBS with 0.25% Triton X‐100 for 1 h at room temperature, followed by primary antibodies incubation overnight at 4°C. Followed by incubation with the horseradish peroxidase (HRP)‐conjugated secondary antibody (Invitrogen, 31460). The colors in all slides were developed by incubation with 3,3N‐Diaminobenzidine Tertrahydrochloride (DAB, Beyotime # P0203), following the manufacturer's guidelines. After that, the slides were stained with haematoxylin (Beyotime # C0107) and mounted in neutral balsam (SCR # 10004160). Images were obtained using Olympus (VS120) microscopy.

For immunofluorescence staining, freshly isolated tumor tissues were fixed in 4% paraformaldehyde for 24 h. Tissues were embedded in OCT, sectioned at thicknesses of 8 µm. Then the sections were washed with PBS twice, blocked with 5% normal goat serum in PBS with 0.25% Triton X‐100 for 1 h at room temperature, followed by primary antibodies incubation overnight at 4°C (CD8a: BioLegend, 100701; CD4: BioLegend, 100505). Followed by incubation with the Alexa Fluor 488‐conjugated secondary antibody (Invitrogen, A11006). After that, the slides were stained with DAPI and mounted (Vectorlabs, H‐1200‐10). Images were obtained using Zeiss LSM 880 confocal.

### 4.10 CAR‐T Cell Production

For human CAR‐T cell production, CD3+ T‐cells were negatively enriched from PBMC (from FuHeng, FH‐H073) using Human CD3+ T Cells Negative Selection Kit (MCE, HY‐K0304). T‐cells were expanded in vitro by culturing in RPMI‐1640 supplemented with 10% heat‐inactivated fetal bovine serum, 1 mm sodium pyruvate (Gibco), 10 mm HEPES (Gibco), 1× MEM non‐essential amino acids (Gibco), 1× penicillin‐streptomycin (Gibco), 50 µM 2‐Mercaptoethanol (Gibco), 100 IU of recombinant IL‐2, and Human CD3/CD28 T cell Activation Beads Kit (Proteintech, KMS310) at a bead:cell ratio of 1:1. 48 and 72 h after expansion, T‐cells were spinoculated (2,000 ×g, 60 min, 30°C) with viral supernatant (anti‐CD19 CAR‐lenti‐virus, the anti‐CD19 CAR plasmid was a gift from HP Wang lab). After the second spinoculation, cells were rested for 2 days and used in experiments.

### 4.11 Luciferase‐Based Killing Assay

Firefly luciferase was induced by lentiviral transduction, and transduced cells were selected with 20 µg/mL of Blasticidin S (Yeasen, 60218ES10). One hundred thousand lymphoma cells expressing the firefly luciferase were pretreated with 20 nm C36 or DMSO for 3 days and then co‐cultured with CAR‐T‐cells at 1:1 effector‐to‐target ratios. 72 h after co‐incubation, cells were transferred into 96‐well white plates (Corning) and mixed with D‐Luciferin (vazyme, DD1208‐01). Luminescence signals were evaluated by a Biotek Synergy Plate Reader.

### 4.12 Next‐Generation Sequencing Data Analysis

Sequences were aligned to the human genome (hg38). All of the methods were used as previously described [62]. RNA‐seq Data Analysis. mRNA levels of genes in triplicate samples were calculated as FPKM. We determined differential gene expression using R package DESeq2 (version v1.20.0) with an FDR threshold of 0.05 and an Log2 Fold Change threshold of ±1.

### 4.13 Enrichment Analysis

Fisher‐exact test. Through Python (version 3.7.0) and its Scipy (version 1.2.1) module, the enrichment level of each gene set was calculated according to Fisher's exact test method.Database. Gene sets download from Molecular Signatures Database (MSigDB, http://www.gsea‐msigdb.org/gsea/msigdb/index.jsp, version v7.2).

### 4.14 Visualization

Data visualization was performed using Python's Matplotlib (version 3.1.0) and Seaborn (version 0.9.0) module, R's Gviz (version 1.30.3), ComplexHeatmap (version 2.12.1), and ggplot2 (version 3.4.0) packages, and DeepTools software (version 3.4.3/3.5.1).

### 4.15 Statistical Analysis and Reproducibility

Statistical analysis for qPCR and tumor tissue section staining was carried out using GraphPad Prism 9. Data are shown as mean ± sd for qPCR data or mean ± sem as noted in the legend. The variance within each group is similar. Statistical significance was determined using the two‐tailed Student's test (t test) or ANOVA test as annotated in the legend. *p* < 0.05 was considered significant. All cellular experiments were repeated multiple (two or more) times with biological repeats.

## Author Contributions

W.Q. and H.Y. conceived and designed the study. T.C. purified the human PRC2 complex; T.C., H.G., and Y.L. performed cryo‐EM grid preparation, data collection and processing, and model building; T.C., Z.Z. and D.L. performed biochemical assays. D.L. performed all cellular studies and xenograft studies with the help from Z.L., D.T., W.Z., and Y.Q. C.Q. performed RNA‐seq and proteomic profiling data analysis. D.L., H.G., H.Y., and W.Q., analyzed the data and wrote the manuscript with input from all authors.

## Funding

This work was supported by grants from the Ministry of Science and Technology (MOST) of China (2023YFA0913404) to W. Q, National Natural Science Foundation of China (No. 92253301 and No. 32270648 to W.Q., No. 32271258 to H.G. and No.32130053 to H.Y), “Pioneer” and “Leading Goose” R&D Program of Zhejiang 2024SSYS0036 to and New Cornerstone Investigator Program to H.Y. This research was also supported by Shanghai Frontiers Science Center for Biomacromolecules and Precision Medicine at ShanghaiTech University.

## Conflicts of Interest

The authors declare no conflicts of interest.

## Supporting information




**Supporting File**: advs76025‐sup‐0001‐SuppMat.docx.

## Data Availability

The cryo‐EM density maps of the complex generated in this study have been deposited to the Electron Microscopy Data Bank under the accession numbers EMD‐62474 (PRC2‐SAH‐H3K27me3), EMD‐62475 (PRC2‐C36), EMD‐62476 (PRC2‐SAH‐H3K27me3‐C36). Atomic coordinates have been deposited to the RCSB Protein Data Bank under the accession numbers 9KOF (PRC2‐SAH‐H3K27me3), 9KOG (PRC2‐C36), 9KOH (PRC2‐SAH‐H3K27me3‐C36). The raw data files for the sequencing analysis that generated in this study are deposited in the NCBI Gene Expression Omnibus (GEO) under the accession numbers: GSE281570.
