# Supplementary material for: Allosteric Inhibition of Polycomb Repressive Complex 2 by an EZH2‐Selective Small Molecule Inhibitor
Source: Adv Sci (Weinh). 2026 Jun 18:e76025. Online ahead of print. doi: 10.1002/advs.76025 (PMC13337107; doi:10.1002/advs.76025)
Supplement: Supplementary file 1 — Supporting File: advs76025‐sup‐0001‐SuppMat.docx. [file ADVS-9999-e76025-s001.docx]

**Supplementary Materials for**

**Allosteric Inhibition of Polycomb Repressive Complex 2 by an EZH2-Selective Small Molecule Inhibitor**

Ting Cao^1,2,†^, Dongdong Liu^3,4,†^, Haishan Gao^1,2, †^, Chenyang Qin^3,5^, Wen Zhang^3^, Ziyun Lu^3^, Dongxia Tan^3^, Yuxiu Qu^3^, Yusong Liu^1,2^, Zhiran Zou^1,2^, Hongtao Yu^1,2,^*, Wei Qi^3,4,5,^*

^1^ Westlake Laboratory of Life Sciences and Biomedicine, School of Life Sciences, Westlake University, Hangzhou, Zhejiang, 310030, China

^2^ New Cornerstone Science Laboratory, Westlake University, Hangzhou, Zhejiang, 310030, China

^3^ Gene Editing Center, School of Life Science and Technology, ShanghaiTech University, Shanghai, 201210, China

^4^ Shanghai Clinical Research and Trial Center, Shanghai, 201210, China

^5^ Lingang Laboratory, Shanghai, 200031, China

This study does not require ethical approval or consent from patients.

† These authors contribute equally to this study.

* Correspondence to: yuhongtao@westlake.edu.cn, [qiwei@shanghaitech.edu.cn](mailto:qiwei@shanghaitech.edu.cn)

**Supplementary Materials and Methods**

**Western blotting**

For western blotting, whole-cell lysates were prepared in 1× SDS loading buffer containing protease inhibitor cocktail (Targetmol, C0001) and boiled at 95 °C for 10 min before loading. Then, 5-20 µl of lysates were separated by SDS-PAGE and transferred to a nitrocellulose membrane using the wet-transfer method. The membranes were blocked with 5% non-fat milk in Tris-buffered saline with 0.1% Tween 20 (TBST) for 1 h at room temperature and then incubated in primary antibodies overnight at 4 °C. The next day, the membranes

were washed in TBST (3×10 min) and then incubated with horseradish

Peroxidase (HRP)-conjugated secondary antibody for 1h at room temperature. After washes with TBST (4×10 min), Pierce ECL substrate was added onto

the membrane and incubated for 2 min before the chemiluminescent signal detection. The following commercial antibodies were used: H3 (CST, 9715), H3K27me3 (CST, 9733), STAT1 (CST, 9172), p-STAT1 (CST, 9167), anti-Flag (Sigma-Aldrich, F1804), CHC (BD, 610500). All primary antibodies were used at a 1:1,000 dilution in 3% BSA in TBST. Secondary antibodies were used at a 1:5,000 dilution in 5% non-fat milk in TBST.

**Quantitative PCR (qPCR) and ChIP-qPCR**

Cells were fixed with 0.8% formaldehyde in culture medium for 10 min at room temperature and the reaction was quenched with 0.125 M glycine sharply. Then remove media and wash the cell twice with cold PBS with protease inhibitor mix. Cells were scraped off the plate, and the pellets were collected, lysed at the volume of 4×10^6^ per 300 μL using SDS lysis buffer for 10 min on ice, and then proceeded to sonication. Each sample was sonicated for 8 min, 15 s on and 45 s off with 80% output (Qsonica 4905 Chiller). Sonicated samples were diluted with ChIP dilution buffer and applied to 1 h pre-clear with 20 μL protein A + G agarose beads. After that, input samples were collected, and every 300 uL of cell lysate samples were applied into ChIP with 1 μg IgG, H3K27me3 and SUZ12 antibody. Antibody-chromatin complexes were incubated overnight at 4 °C, then captured by 60 μL protein A + G agarose beads for 1 h at 4 °C. The beads were washed in low salt buffer once, high salt buffer once, LiCl buffer once, and TE buffer twice. DNA-protein complex was eluted twice with elution buffer (1% SDS, 0.1 M NaHCO_3_), eluted chromatin was de-crosslink at 65 °C for 4 h with 5 M NaCl and 2 μL Proteinase K. Reserved chromatin DNA were purified with Phenol-chloroform method.

**The colony forming cell (CFC) assay**

Thaw aliquots of methylcellulose-based medium (Stem cell, 03534) at room temperature for approximately 30 minutes. Allow the vials to thaw without disturbance. During the thaw step, resuspend the mouse bone marrow cell sample in 10 mL of IMDM/2% FBS or in an appropriate volume and count. Centrifuge for 8 minutes at 300 g. Remove the supernatant and resuspend the cells in IMDM/2% FBS or the appropriate medium to the desired cell concentration for plating. Cell samples should be resuspended in IMDM/2% FBS. The culture system was established in a total volume of 3.3 mL, consisting of methylcellulose-based medium supplemented with 300 μL cell suspension (final cell number: 1*10^4^/1.1 mL), a gradient of PRC2 inhibitor (final concentrations: 5, 1, 0.2, 0.04, 0.008, and 0.0016 μM, with a DMSO control), and 2 μM Cisplatin (MCE, HY-17394) as a positive control. Vigorously vortex the vial to thoroughly mix the cells with the media. Wait for approximately 20 minutes before continuing with the procedure to allow air bubbles to escape. Add 1.1 mL of the final cell mixture into 35 mm culture dish (non-tissue culture treated petri dishes) using a 3 mL syringe fitted with a 16-gauge needle. Spread the media evenly by gently rotating the dish. Place two sample dishes and an uncovered dish containing 3-4 mL of sterile water in a 100 mm culture dish and cover. The sterile water dish serves to maintain the humidity necessary for colony development. Incubate the cells for 12 days at 37 degree and 5% CO2. Avoid disturbing the dish during the incubation period to prevent shifting of the colonies. Score colonies at the end of the incubation period. Identify and count individual colonies using an inverted microscope and a scoring grid.

**CCK8 assay**

Dispense 100 μL of cell suspension (10000 cells/well) in a 96-well plate. Add a gradient of PRC2 inhibitor (final concentrations: 10, 2, 0.4, 0.08, and 0.016 μM, with a DMSO control), and 2 μM Cisplatin (MCE, HY-17394) as a positive control to the plate. Incubate the plate for 6 days in the incubator at 37°C, 5% CO2. Add 10 µL of CCK-8 solution to each well of the plate. Be careful not to introduce bubbles to the wells, since they interfere with the O.D. reading. Incubate the plate for 4 hours. Measure the absorbance at 450 nm using a microplate reader.


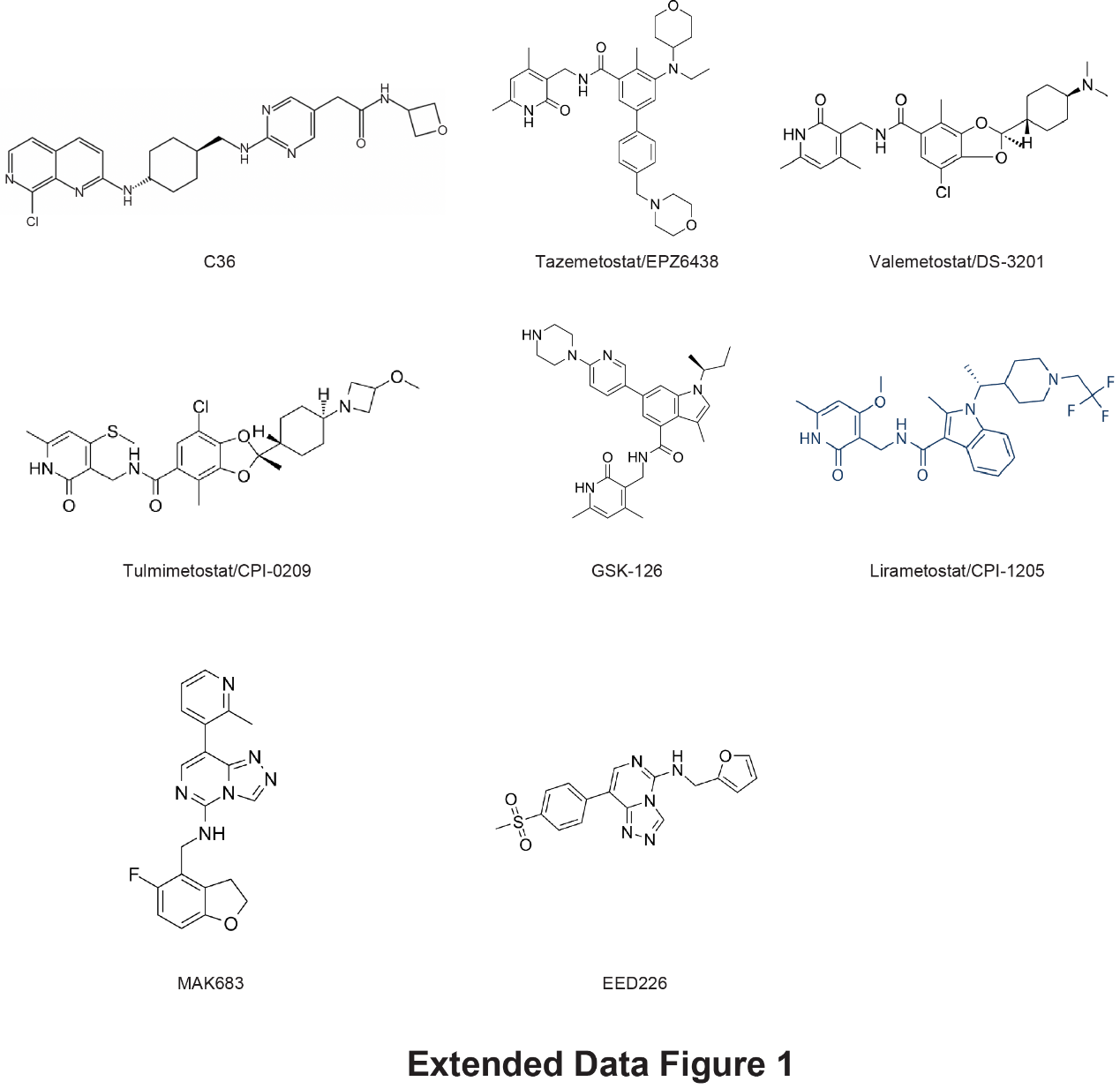


**Figure S1.** **The EZH2 inhibitors mentioned in this study.**


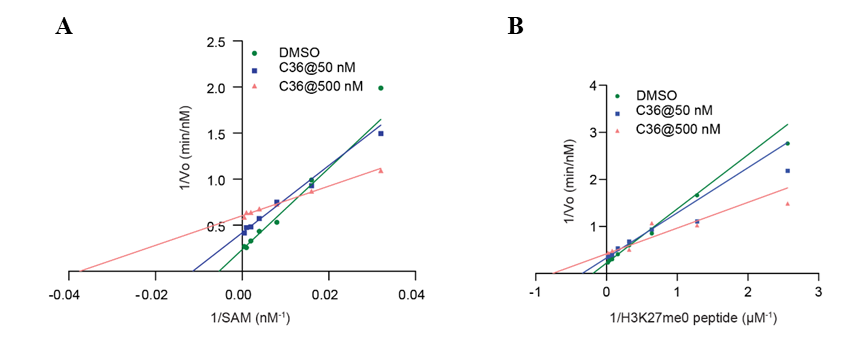


**Figure S2.** **C36 inhibition on PRC2 in the presence of SAM or H3K27me0 peptide. (A)** The Lineweaver-Burk plots of C36 inhibition on PRC2 in the presence of SAM. **(B)** The Lineweaver-Burk plots of C36 inhibition on PRC2 in the presence of H3K27me0 peptide.


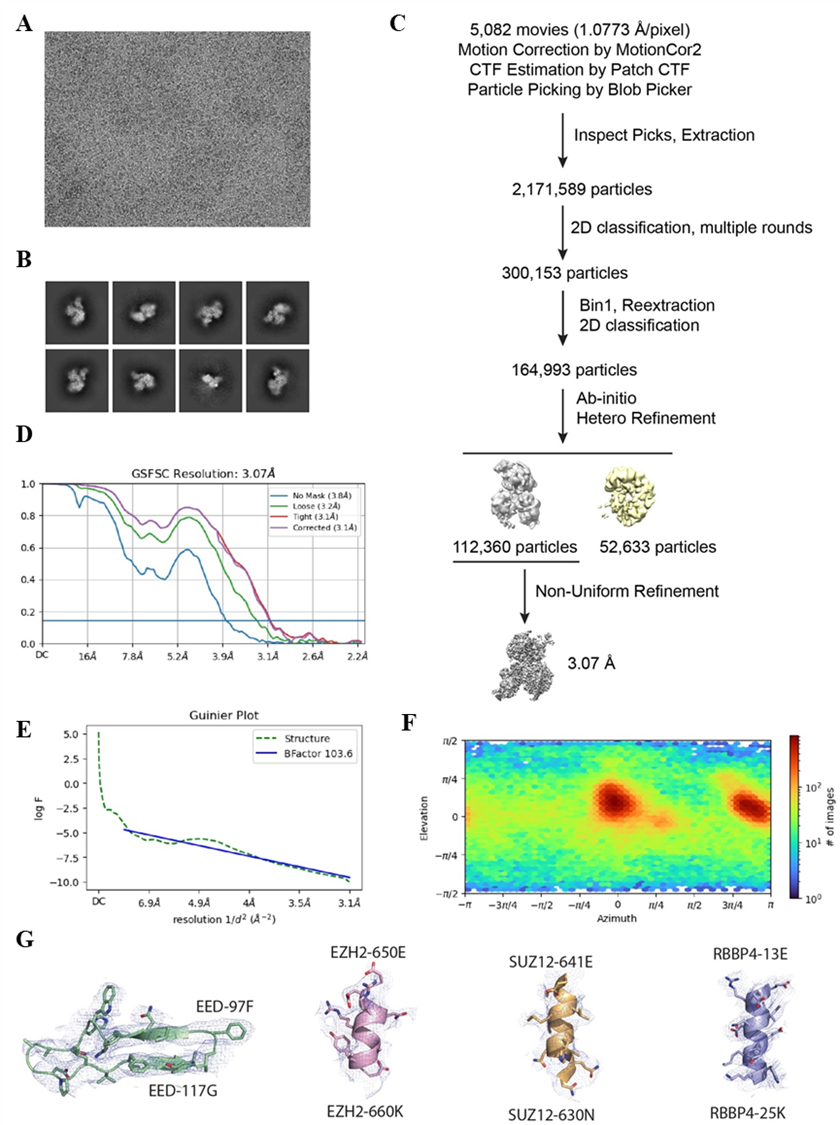


**Figure S3.** **Cryo-EM analysis of PRC2-SAH-H3K27me3 complex. (A)** Representative Cryo-EM micrograph of human PRC2- SAH-H3K27me3 complex. **(B)** Representative 2D class averages of human PRC2-SAH-H3K27me3 complex. **(C)** Cryo-EM image processing flowchart for human PRC2-SAH-H3K27me3 complex. **(D)** The Gold-standard FSC curve of the final EM map for human PRC2-SAH-H3K27me3 complex. **(E)** The Guinier Plot of the final EM map for human PRC2-SAH-H3K27me3 complex. **(F)** The angular distribution of particles used in the final reconstruction for human PRC2-SAH-H3K27me3 complex. **(G)** Representative cryo-EM density in the map of PRC2-SAH-H3K27me3 complex.


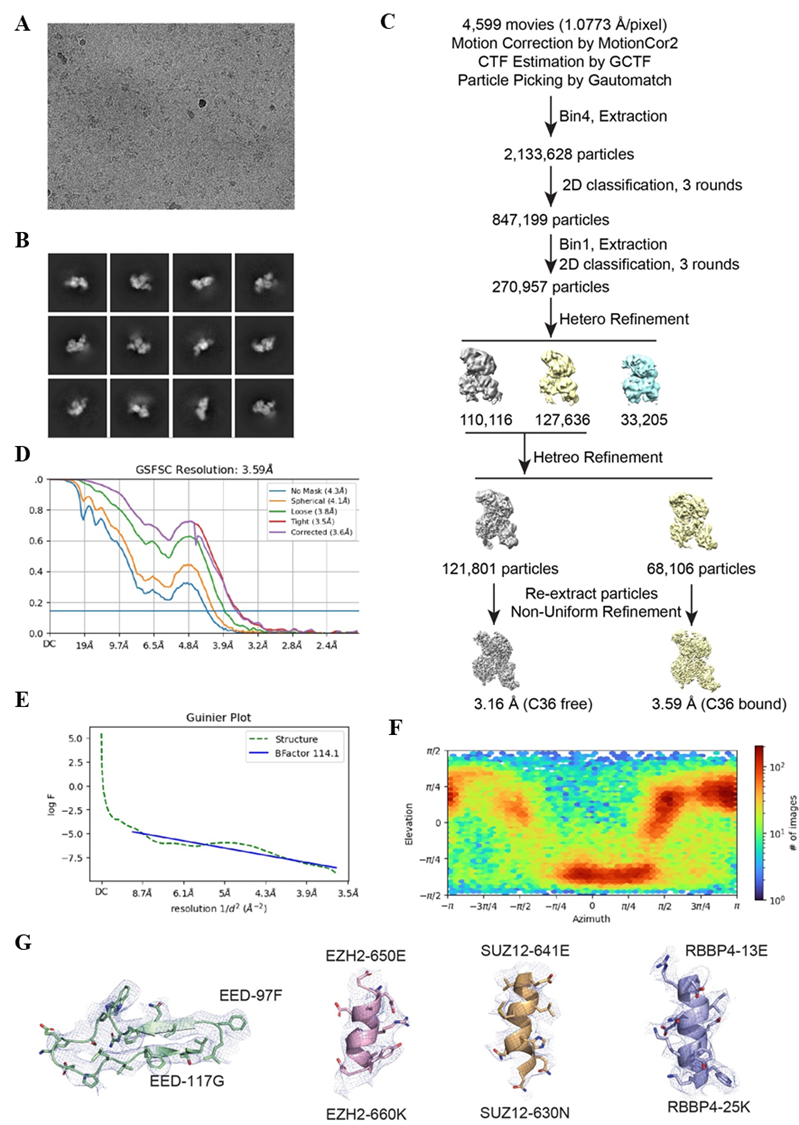


**Figure S4.** **Cryo-EM analysis of PRC2-C36 complex. (A)** Representative Cryo-EM micrograph of human PRC2-C36 complex. **(B)** Representative 2D class averages of human PRC2-C36 complex. **(C)** Cryo-EM image processing flowchart for human PRC2-C36 complex. **(D)** The Gold-standard FSC curve of the final EM map for human PRC2-C36 complex. **(E)** The Guinier Plot of the final EM map for human PRC2-C36 complex. **(F)** The angular distribution of particles used in the final reconstruction for human PRC2-C36 complex. **(G)** Representative cryo-EM density in the map of PRC2-C36 complex.


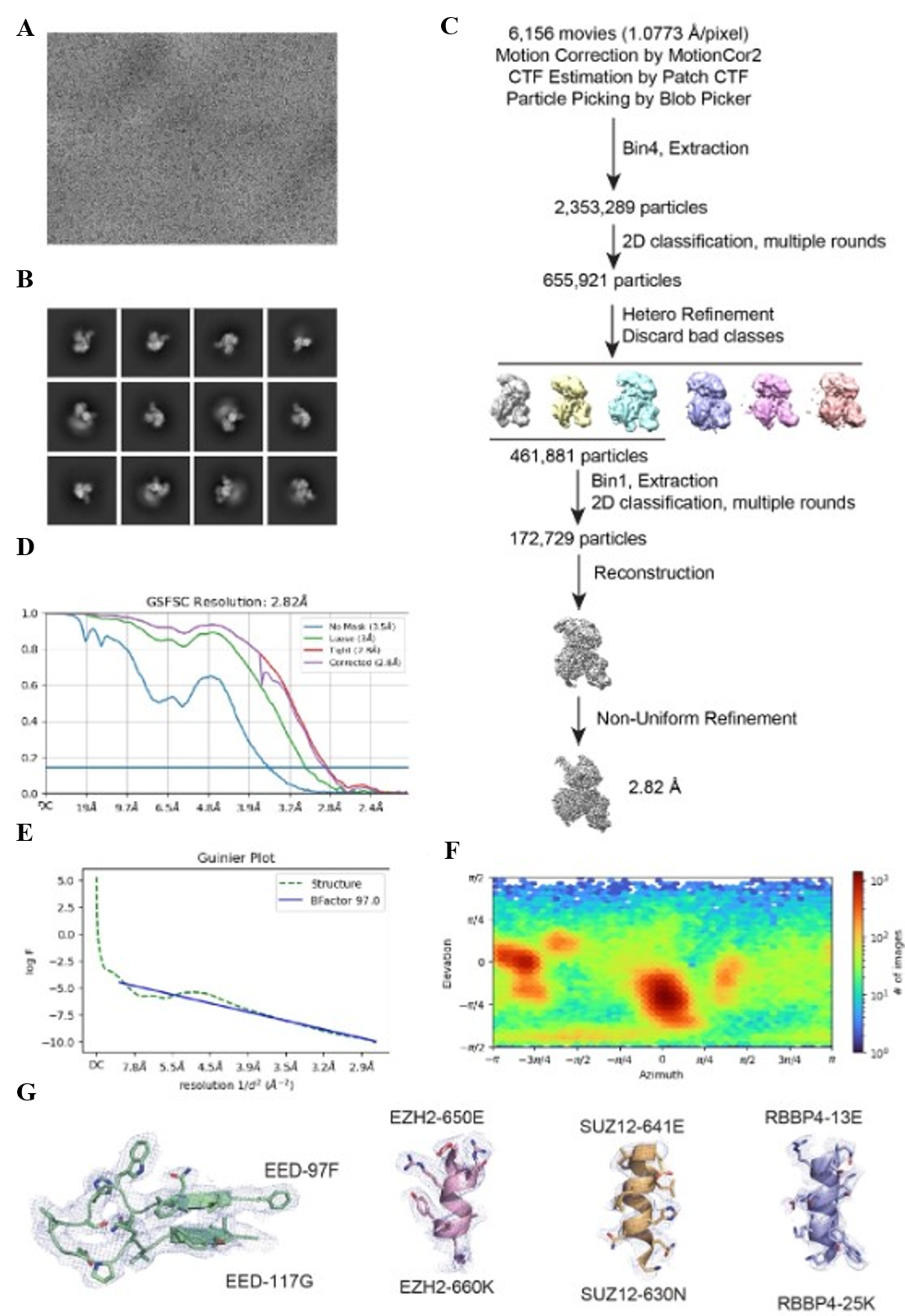


**Figure S5.** **Cryo-EM analysis of PRC2-SAH-H3K27me3-C36 complex. (A)** Representative Cryo-EM micrograph of human PRC2- SAH-H3K27me3-C36 complex. **(B)** Representative 2D class averages of human PRC2-SAH-H3K27me3-C36 complex. **(C)** Cryo-EM image processing flowchart for human PRC2-SAH-H3K27me3-C36 complex. **(D)** The Gold-standard FSC curve of the final EM map for human PRC2-SAH-H3K27me3-C36 complex. **(E)** The Guinier Plot of the final EM map for human PRC2-SAH-H3K27me3-C36 complex. **(F)** The angular distribution of particles used in the final reconstruction for human PRC2-SAH-H3K27me3-C36 complex. **(G)** Representative cryo-EM density in the map of PRC2-SAH-H3K27me3-C36 complex.

**
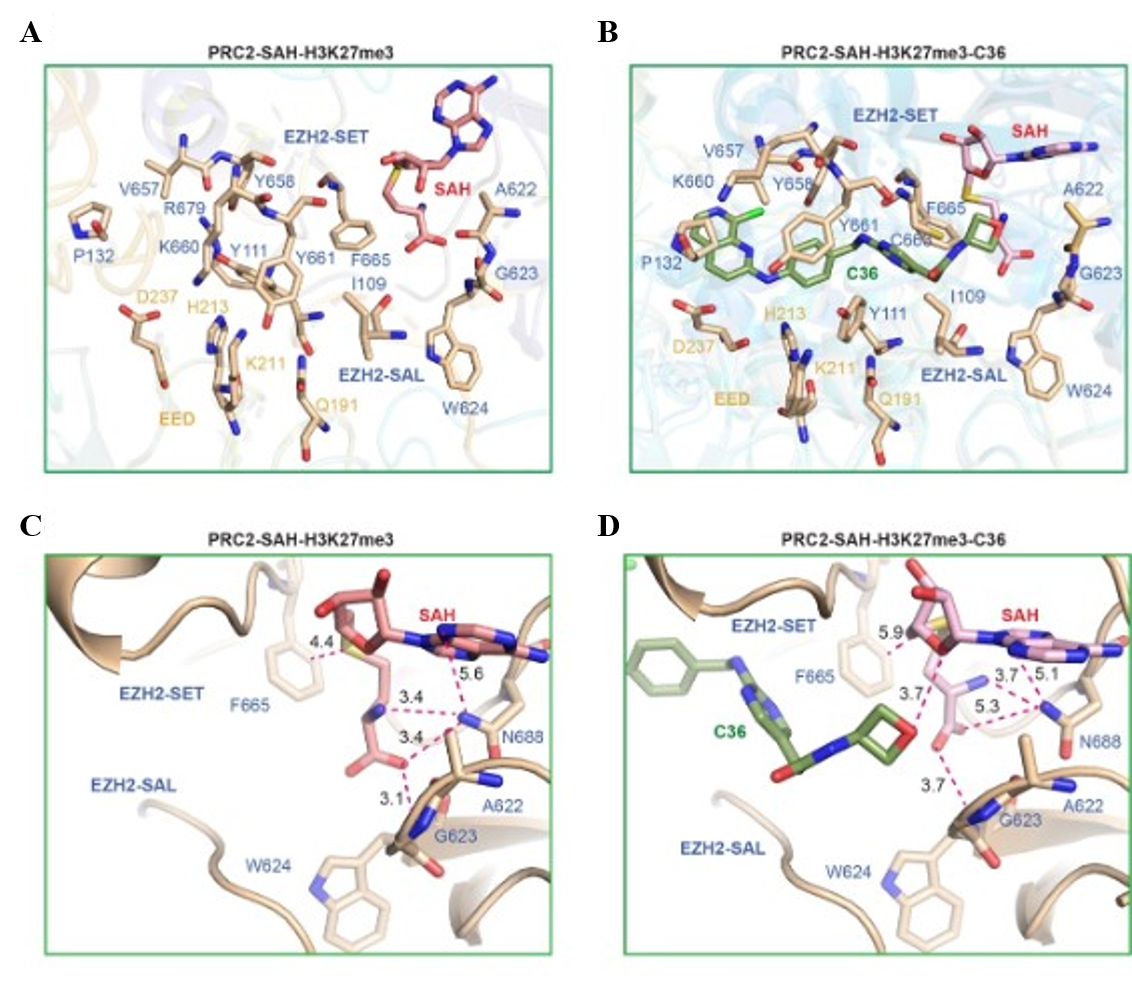
**

**Figure S6.** **C36 binding induces local conformational remodeling in PRC2. (A)** The binding pocket in the structure of PRC2-SAH-H3K27me3 without C36. **(B)** The binding pocket in the structure of PRC2-SAH-H3K27me3 with C36. **(C)** The SAH binding pocket in PRC2-SAH-H3K27me3 without C36. The distances between SAH and its surrounding residues are highlighted. **(D)** The SAH binding pocket in PRC2-SAH-H3K27me3 with C36. The distances between SAH and its surrounding residues are highlighted.


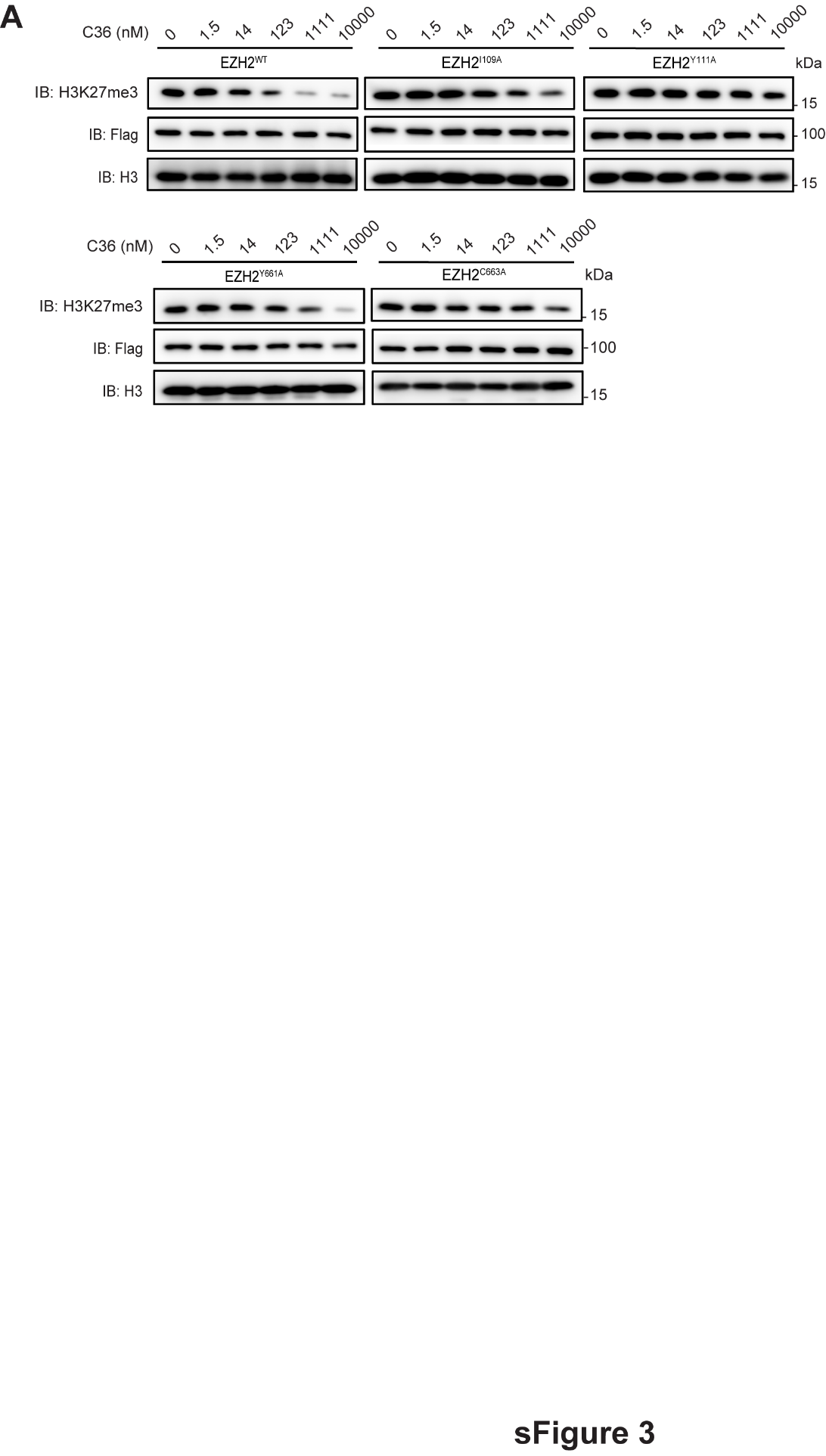


**Figure S7.** **Inhibition mechanism of EZH2/PRC2 by C36. (A)** Western blots of H3K27me3 or the indicated proteins in G401 cells treated with a series dilution of C36 for 3 days. The G401 cells express EZH2 with I109A, Y111A, Y661A or C663A mutations vector and wild type. Repeated 3 times.

**
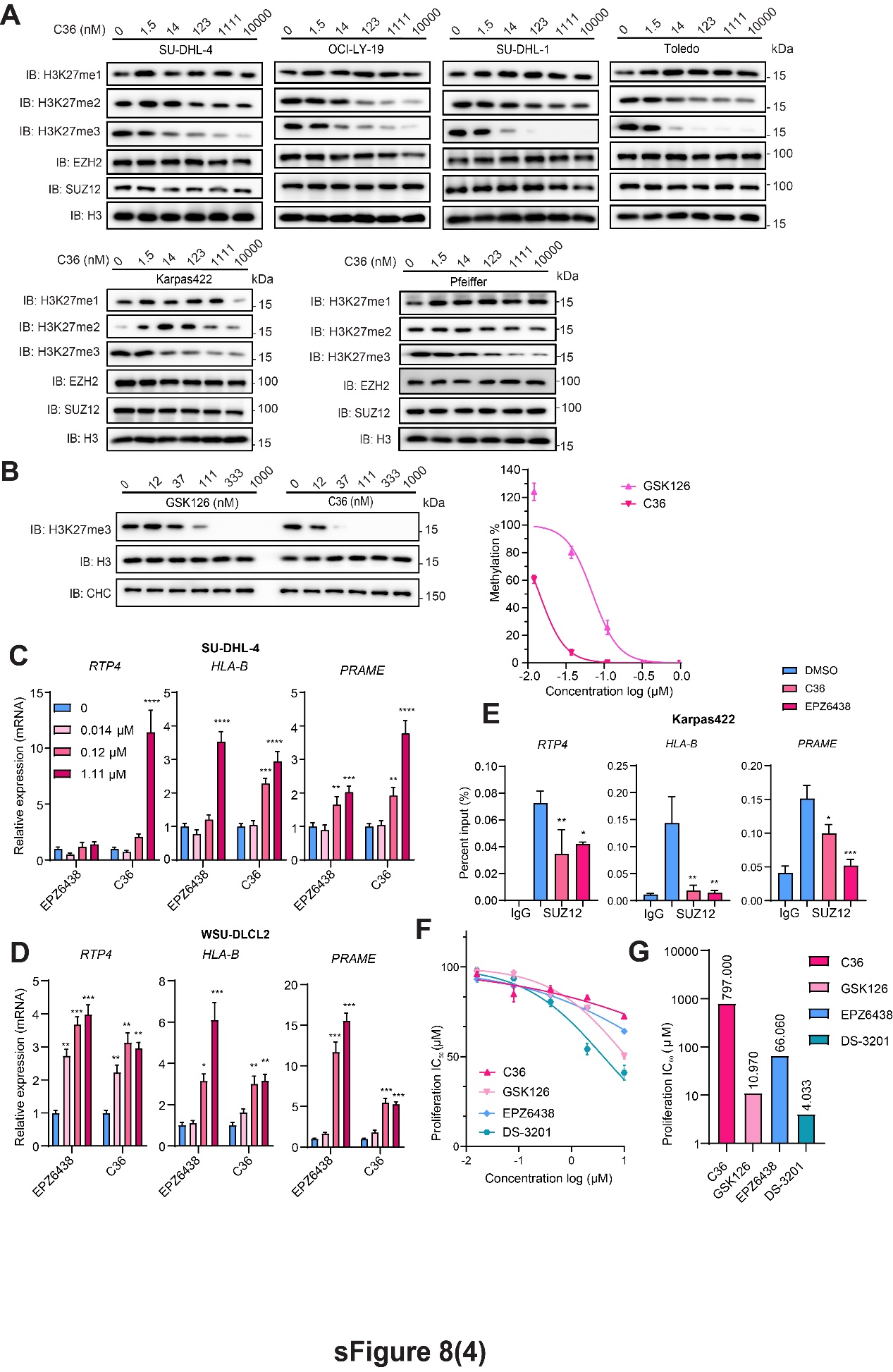
**

**Figure S8.** **C36 inhibits PRC2 in cells. (A)** Inhibition of H3K27 methylation by different concentrations of C36 measured by Western blot. SU-DHL-4, OCI-LY-19, SU-DHL-1, Toledo, Karpas422 and Pfeiffer cells were treated with C36 for 3 days at the indicated concentrations. Repeated 3 times. **(B)** Inhibition of H3K27me3 by different concentrations of C36 (IC_50_ = 14.16 nM) and GSK-126 (IC_50_ = 70.49 nM) measured by Western blot. G401 cells were treated with C36 or GSK-126 for 3 days at the indicated concentrations. Repeated 3 times. **(C** and **D)** Gene expression was determined by RT-qPCR following 5 days treatment with C36 or EPZ6438 in SU-DHL-4 cells and WSU-DLCL2 cells (mean ± s.d., n=3). **(E)** ChIP-qPCR showed SUZ12 at the targeted gene promoters in Karpas422 from **(D)**. Rabbit IgG (Vehicle sample) was used as control (mean ± s.d., n = 4). (**F**) Inhibition of mouse bone marrow cell proliferation was measured by CCK-8 assay following 6-day treatment with the compound at indicated concentrations (0.016, 0.08, 0.4, 2, and 10 μM) or DMSO control. (n≥3). (**G**) Effects of PRC2 inhibitors on proliferation of mouse bone marrow cell by CCK8 assay. The mouse bone marrow cells were treated as (**F**). The calculated IC₅₀ values for each inhibitor are indicated above the respective bars in the graph.Statistical analysis was performed using two-tailed unpaired t test (*, p <0.05; **, p < 0.01; ***, p < 0.001; ****, < 0.0001).

**
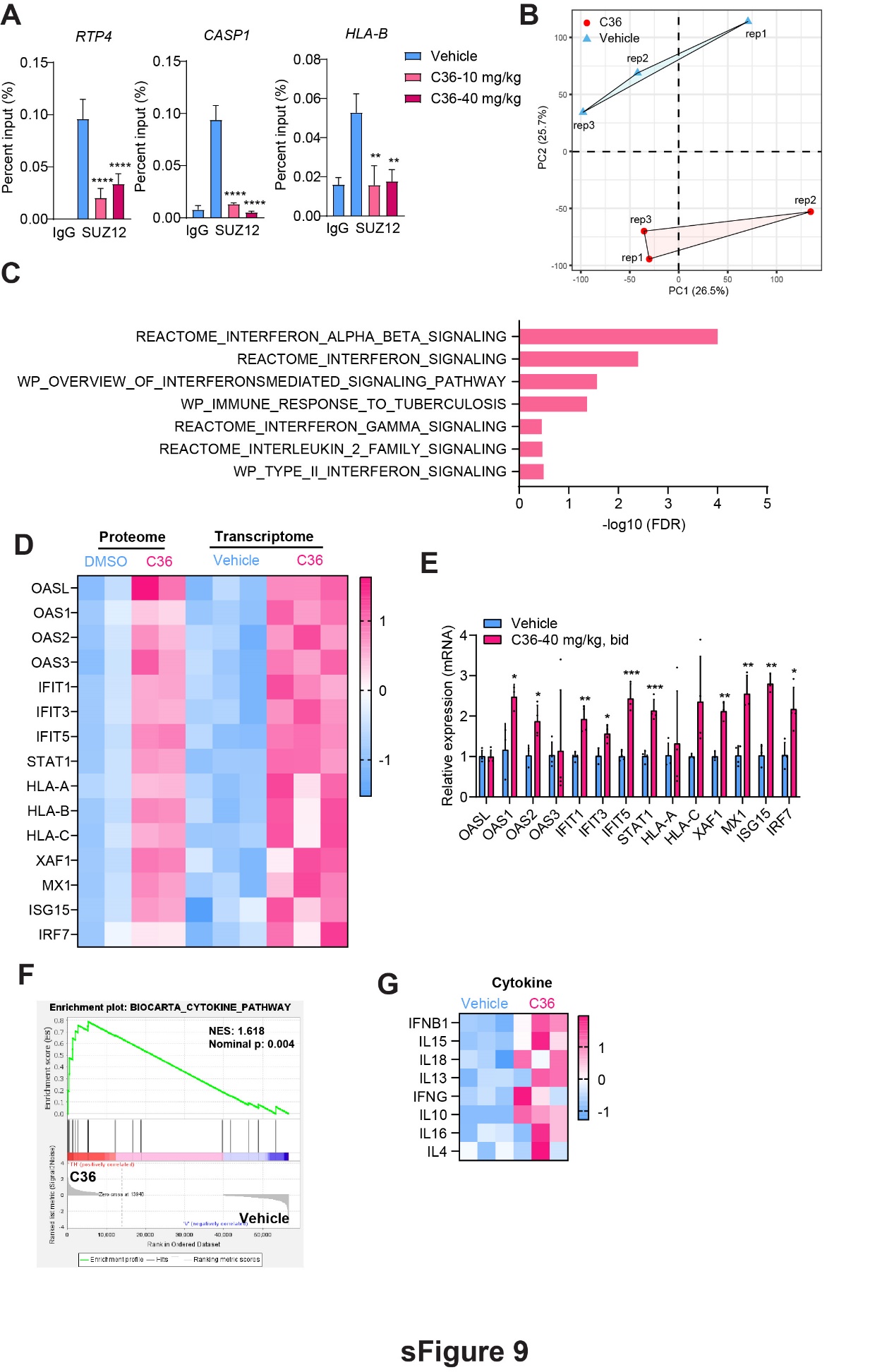
**

**Figure S9.** **C36 treatment triggers interferon signaling. (A)** ChIP-qPCR showed SUZ12 at the targeted gene promoters in tumor samples from (Fig. 5D). Rabbit IgG (Vehicle sample) was used as control (mean ± s.d., n = 4). Statistical analysis was performed using one-way ANOVA (*, p <0.05; **, p < 0.01; ***, p < 0.001; ****, < 0.0001). **(B)** and **(C)** PCA plot of RNA-seq and the top enriched reactome pathways by RNA-seq in Fig. 6A**. (D)** Heatmap depicting the top enriched gene expression associated with the interferon signaling pathway, as shown in Fig. 6D. **(E)** Gene expression associated with interferon signaling pathways, as shown in Fig. 6D, was determined by RT-qPCR. Tumor samples were from the end point of study in Fig. 5D (mean ± s.d., n = 4 mice per group). Statistical analysis was performed using two-tailed unpaired t test (*, p <0.05; **, p < 0.01; ***, p < 0.001; ****, < 0.0001). **(F)** GSEA analysis showing the enrichment of Biocarta_cytokine_Pathway in the C36 treated tumor samples. **(G)** Heat map depicting the gene expression associated with the cytokine, as shown in (F).

**
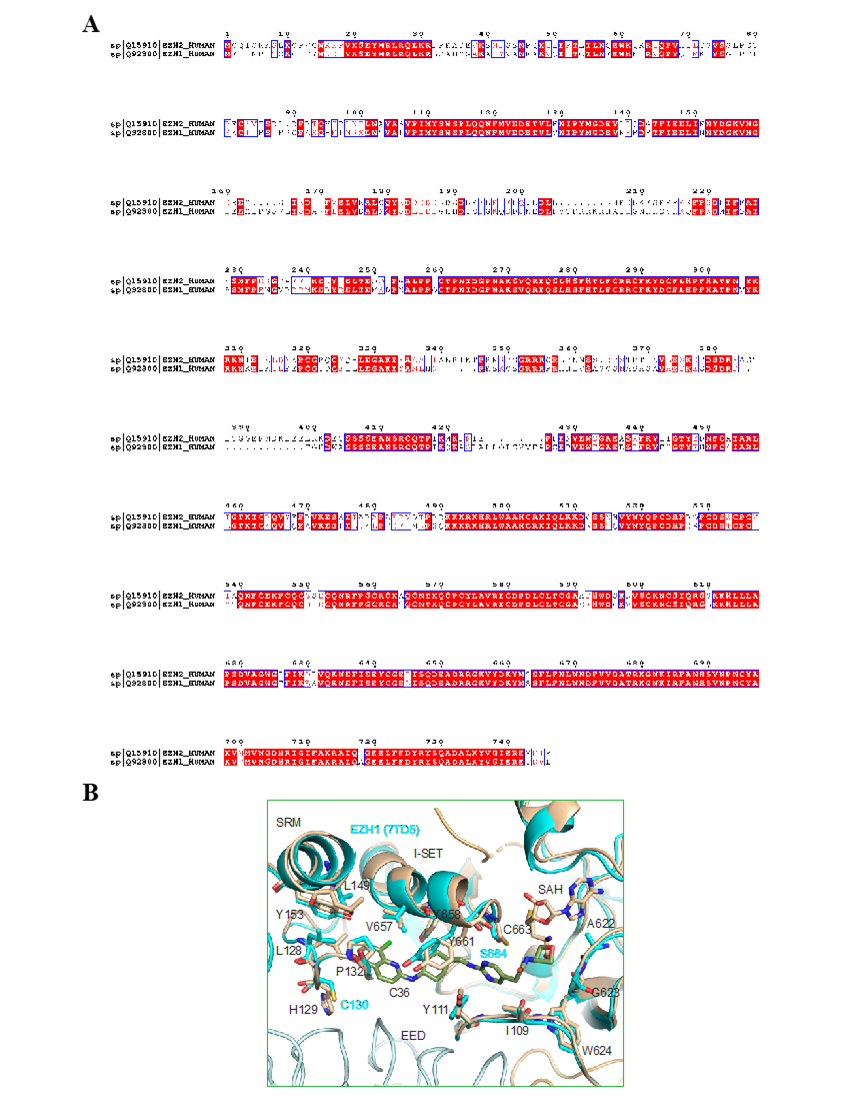
**

**Figure S10.** **Structural basis for the selectivity of C36 on EZH2 over EZH1. (A)** The amino acid sequence alignment between human EZH2 and EZH1. The alignment is generated by Clusta Omega and shown by ESPript 3.0. The amino acids number is assigned according to EZH2. **(B)** Structural comparison between EZH1 (colored in cyan, PDB code 7TD5) and C36 bound EZH2 (colored in light orange).

**
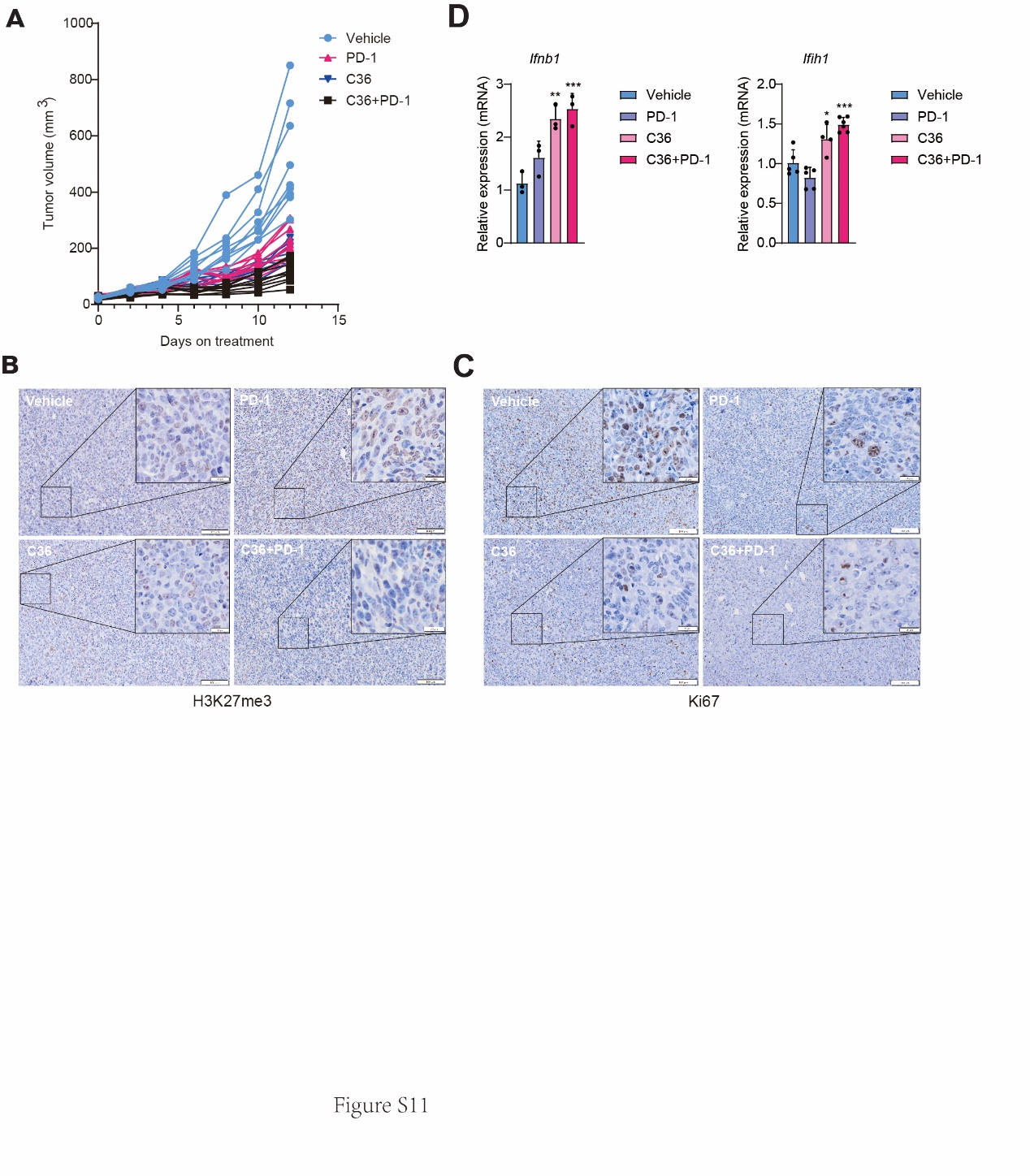
**

**Figure S11. Combined treatment with C36 and PD-1 improves anti-tumor immune responses.**

(**A**) Growth curve of subcutaneous LLC tumors in C57Bl/6 mice treated with twice-daily oral C36 (30 mg kg−1) or vehicle and subcutaneous anti-PD-1 (10 mg kg−1) or PBS. (**B**) and (**C**) Representative H3K27me3 and Ki67 IHC images of tumor samples from the end point of study in (Figure7D). Scale bar for images, 100 μm; scale bar for the intersects, 20 μm. (**D**). Gene expression was determined by RT-qPCR, tumor samples from the end point of study in (Figure7D) (mean ± s.d., n ≥ 3).

**Table S1. Cryo-EM data collection, refinement, and validation statistics**

|  | **PRC2-SAH-H3K27me3** | **PRC2-SAH-H3K27me3-C36** | **PRC2-C36** |
| --- | --- | --- | --- |
| **Data Collection and Processing** | | | |
| Microscope | FEI Titan Krios  105,000  300  50  Gatan K3 Summit  -1.0 to -2.0  1.0773 | | |
| Magnification |  |  |  |
| Voltage |  |  |  |
| Electron dose (e^-^/Å^2^) |  |  |  |
| Detector |  |  |  |
| Defocus range (um) |  |  |  |
| Pixel size (Å/pixel) |  |  |  |
| Micrographs (no.) | 5,082 | 6,156 | 4,599 |
| Initial particles (no.) | 2,716,189 | 2,927,647 | 2,133,628 |
| Final particles (no.) | 112,360 | 172,729 | 67,922 |
| Symmetry imposed | C1 | C1 | C1 |
| Map resolution (Å) | 3.07 | 2.82 | 3.59 |
| FSC threshold | 0.143 | 0.143 | 0.143 |
| **Model composition** | | | |
| Protein residues | 1676 | 1730 | 1752 |
| Ligands | SAH | C36, SAH | C36 |
| **Refinement** | | | |
| Initial model used  (PDB code) | 5WG6, 5HYN | 5WG6, 5HYN | 5WG6, 5HYN |
| Model resolution (Å) | 3.3 | 3.1 | 3.8 |
| FSC threshold | 0.143 | 0.143 | 0.143 |
| Map sharpening B factor (Å^2^) | 104 | 97 | 114 |
| **Validation** | | | |
| MolProbility score | 2.25 | 2.11 | 2.13 |
| Clash score | 16.06 | 12.44 | 12.57 |
| Rotamer outliers (%) | 0.14 | 0 | 0.13 |
| C_β_ outliers (%) | 0.31 | 0 | 0 |
| **R.m.s deviations** | | | |
| Bonds length (Å) | 0.004 | 0.004 | 0.003 |
| Bonds Angle (°) | 0.857 | 0.710 | 0.585 |
| **Ramachandran plot (%)** | | | |
| Favored | 90.20 | 91.38 | 90.98 |
| Allowed | 9.25 | 8.14 | 8.85 |
| Outliers | 0.55 | 0.48 | 0.17 |
